# Future Coastal Population Growth and Exposure to Sea-Level Rise and Coastal Flooding - A Global Assessment

**DOI:** 10.1371/journal.pone.0118571

**Published:** 2015-03-11

**Authors:** Barbara Neumann, Athanasios T. Vafeidis, Juliane Zimmermann, Robert J. Nicholls

**Affiliations:** 1 Institute of Geography, Kiel University, Kiel, Germany; 2 Faculty of Engineering and the Environment and Tyndall Centre for Climate Change Research, University of Southampton, Southampton, United Kingdom; University of New England, AUSTRALIA

## Abstract

Coastal zones are exposed to a range of coastal hazards including sea-level rise with its related effects. At the same time, they are more densely populated than the hinterland and exhibit higher rates of population growth and urbanisation. As this trend is expected to continue into the future, we investigate how coastal populations will be affected by such impacts at global and regional scales by the years 2030 and 2060. Starting from baseline population estimates for the year 2000, we assess future population change in the low-elevation coastal zone and trends in exposure to 100-year coastal floods based on four different sea-level and socio-economic scenarios. Our method accounts for differential growth of coastal areas against the land-locked hinterland and for trends of urbanisation and expansive urban growth, as currently observed, but does not explicitly consider possible displacement or out-migration due to factors such as sea-level rise. We combine spatially explicit estimates of the baseline population with demographic data in order to derive scenario-driven projections of coastal population development. Our scenarios show that the number of people living in the low-elevation coastal zone, as well as the number of people exposed to flooding from 1-in-100 year storm surge events, is highest in Asia. China, India, Bangladesh, Indonesia and Viet Nam are estimated to have the highest total coastal population exposure in the baseline year and this ranking is expected to remain largely unchanged in the future. However, Africa is expected to experience the highest rates of population growth and urbanisation in the coastal zone, particularly in Egypt and sub-Saharan countries in Western and Eastern Africa. The results highlight countries and regions with a high degree of exposure to coastal flooding and help identifying regions where policies and adaptive planning for building resilient coastal communities are not only desirable but essential. Furthermore, we identify needs for further research and scope for improvement in this kind of scenario-based exposure analysis.

## Introduction

Coastal zones have always attracted humans because of their rich resources, particularly their supply of subsistence resources; for logistical reasons, as they offer access points to marine trade and transport; for recreational or cultural activities; or simply because of their special sense of place at the interface between land and sea. The development and utilisation of coastal zones has greatly increased during the recent decades and coasts are undergoing tremendous socio-economic and environmental changes—a trend which is expected to continue in future. Further, coastal areas show distinctive patterns of population structures and development, which are partially linked to the global trends of growth and urbanisation. Population density is significantly higher in coastal than in non-coastal areas [1, 2] and there is an ongoing trend of coastal migration, which is associated with global demographic changes [3]. Coastal population growth and urbanisation rates are outstripping the demographic development of the hinterland, driven by rapid economic growth and coastward migration [4, 5]. In China and Bangladesh, for example, the population in the low-elevation coastal zone (LECZ) grew at around twice the rate of the national growth between 1990 and 2000 [5]; the LECZ is commonly defined as the contiguous and hydrologically connected zone of land along the coast and below 10 m of elevation [5, 6]. At the same time, urban areas in the LECZ are growing and expanding faster than in any other area [7]. In China, the growth of coastal urban areas is particularly high at more than three times the national rate, which has been associated with the on-going economic development and specific policies that drive coastward migration [5].

Most of the world’s megacities are located in the coastal zone [8] and many of these are situated in large deltas, where combinations of specific economic, geographic and historical conditions to date attract people and drive coastal migration [9]. This trend, however, is not restricted to mega-deltas: de Sherbinin et al. [10] estimate that globally nearly all coastal ecosystems, as categorised by the Millennium Ecosystem Assessment, experienced net in-migration between 1970 and 2000 despite prevalent coastal hazards. Further, as observed by Seto et al. [7] in a global meta-analysis of urban land-use change, urban land expansion rates in the coastal zone were significantly higher than in the non-coastal hinterland in the same period. These trends are commonly assumed to continue into the future or to even increase [7, 11, 12], making this an important scenario to consider in policy analysis [13]. However, coastal population growth and urbanisation trends are not uniform and can vary significantly between countries and regions: The highest rates of urban land conversion in the coastal zone, i.e. increase of urban extent, occurred in China and Southwest Asia, while the lowest change took place in Europe, North America and Oceania [7].

Population growth and development are critical drivers of change in coastal zones and generate a high pressure on coastal ecosystems and natural resources through increased utilisation and pollution [14, 15]. Coastal growth, land conversion and urbanisation are also related to an increasing exposure of large numbers of people and assets to existing hazards and sea-level rise and related effects, which significantly increases levels of risk and vulnerability along coastlines and in populated deltas. This holds especially true for countries of the developing world [16–18]. Changes in extreme coastal high water levels due to climate change and sea-level rise and the biophysical and socio-economic consequences of such hazards could render living at the coast a high-risk choice [16, 19–21]. Recent studies suggest that mean sea levels could rise by 1 m or more by 2100 [22, 23], which will have severe impacts on coastal environments and ecosystems. Human coastal settlements including infrastructure and economies could be severely impacted by inundation and flooding, coastal erosion, shoreline relocation or saltwater intrusion; and there is the potential for larger disasters [8, 24, 25]. Furthermore, high-impact coastal hazards, such as tsunamis, can devastate whole regions and result in high casualties, as observed during the 2004 Indian Ocean Tsunami and the Great Eastern Earthquake and Tsunami which hit the northeast coast of Japan in 2011 [20, 26].

At global to regional scales, various studies estimated the population living in the LECZ [1, 5]; assessed the coastal population possibly impacted by a certain rise in sea level [27, 28]; and identified the people living in the storm surge hazard zone that is subject to re-occurring coastal flood events with a specific return rate, with or without consideration of climate change and sea-level rise [18, 29, 30], and adaptation [13, 31–33]. These studies use a range of recognised metrics while working at different spatial and temporal scales and employing various methodological approaches from simple inundation models to more complex vulnerability assessment tools. For reviews of these and other studies and for summaries of commonly employed metrics, data and methods, we refer to Lichter et al. [6], McLeod et al. [34], Mondal and Tatem [35] and Nicholls et al. [36].

The above mentioned studies also differ in the base data used and the scenarios employed. For example, Dasgupta et al. [28, 30] assessed the population of developing countries exposed to sea-level rise and storm surges on the basis of spatially explicit but static population data. Nicholls [13] considered two scenarios of coastal population change in a scenario-based analysis of coastal flooding impacts for the 21^st^ century: First a low-growth scenario, where coastal change was assumed to uniformly follow national change. Second a high-growth scenario, where the coastal population was assumed to grow at twice the rate of the national population in the event of growth, or to decrease at half the rate if declining trends occurred, i.e. people are being relatively attracted to the coast even in the case of falling national population trends. Nicholls et al. [11] tested scenario-driven variations of this “migration factor” with values ranging between one and two and assumed coastward migration to potentially offset falling population trends beyond 2050 for A1 and B1 Special Report on Emissions Scenarios (SRES), resulting in a net increase of population exposed to coastal hazards. Both studies did not differentiate between urban and non-urban population shares.

In this study, we provide more detailed assessments of future coastal population exposure, including accounting for the observed differential growth of coastal areas against the land-locked hinterland, as well as for urbanisation trends and the expansive growth of coastal urban areas [37]. Our key assumption is that the observed trends of coastal growth are likely to continue into the future. We use spatially explicit methods and publicly available global data sets to assess (i) the land area and population distribution in the LECZ and (ii) people living in the 100-year flood plain for three points in time: For a baseline year (2000) and for the years 2030 and 2060. In this context, we develop national projections of the urban and non-urban coastal population on the basis of four environmental and socio-economic scenarios which account for sea-level rise (for the flood plain analysis), population distribution, trends in urbanisation and coastal population growth. Our projections of the LECZ population refer to the extent of LECZ in the baseline year 2000 and do not consider possible displacement due to sea-level rise and other hazards or environmental changes. Further, we apply specific correction factors to account for coastal growth. The underlying scenario narratives, which were developed by the UK Government’s Foresight project on Migration and Global Environmental Change (henceforth the Foresight Project), specifically aim at representing possible future developments of migration drivers [38, 39].

This paper is structured as follows: The **Material and Methods** outline the metrics and methodology chosen, the spatial and demographic base data employed and the projections developed. In the **Results** section, we present the findings for population development in the LECZ and the 100-year flood plain, while in the **Discussion** specific issues are addressed such as scenarios of population development and drivers of coastal migration, as well as limitations and uncertainties. Finally, the **Summary and Conclusions** summarize the study results, which present new estimates of coastal population trends and exposure and build ground for further and more detailed assessments of exposure and vulnerability of coastal zones.

## Material and Methods

There is no uniform definition of the coastal zone. Generally understood as the broader transitional area between the land and the marine environment [40], any geographical delimitation of the “coastal zone” is linked to the questions asked and the specifications of localities and issues under investigation. In the present study, we employed the concept of the LECZ, which constitutes an unambiguous and widely used definition of the coastal zone [5, 6] (see Introduction). In addition to the LECZ metrics, we also used the 100-year flood plain in order to better understand present and future risk. The 1-in-100-year return period is the standard used for coastal protection in many countries and has been employed in many earlier assessments, e.g. in Hanson et al. [18] and Hallegatte et al. [41].

**Fig 1:**
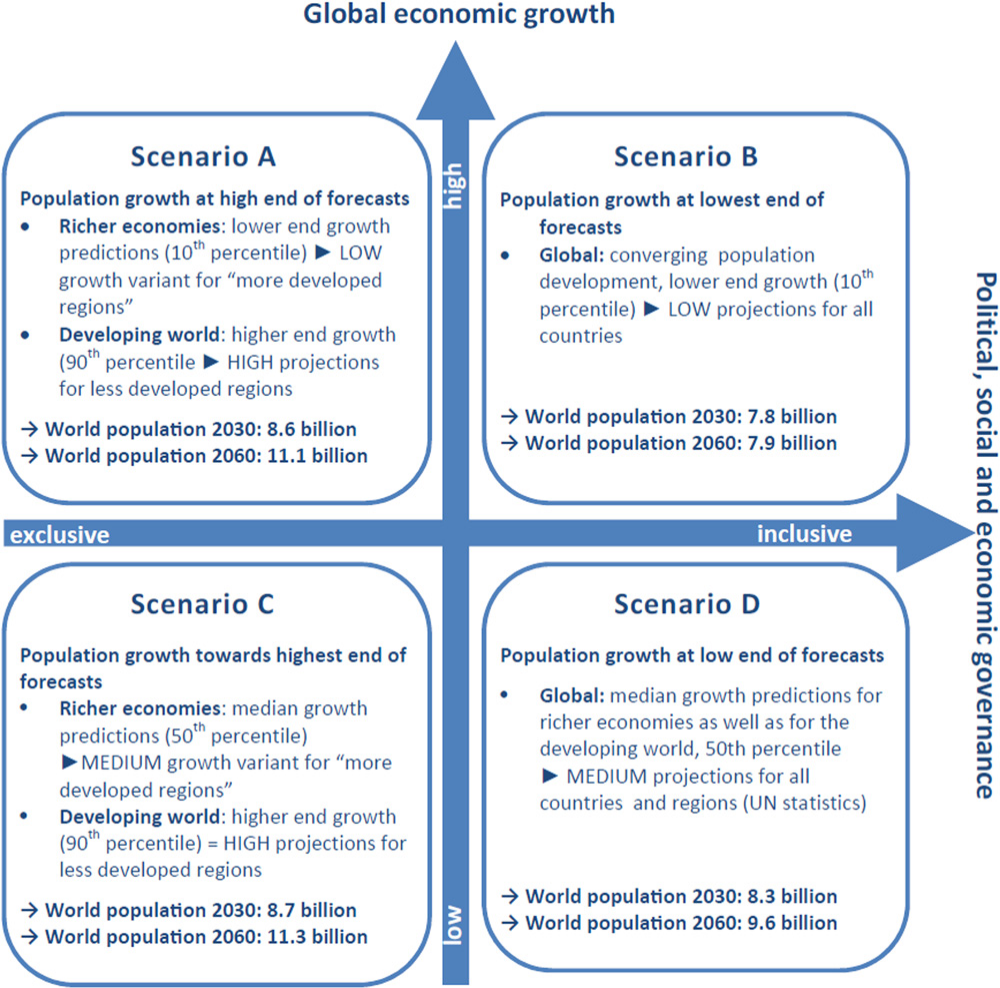
Foresight scenarios A-D of future population growth and implementation through UN demographic variants. Assumptions of future population growth for the Foresight scenarios A-D were taken from [38, 39]. Included in this figure are global scenario results which are based on UN variants of population growth (‘LOW’, ‘MEDIUM’, ‘HIGH’) [46–48] as well as development status.

The population projections for 2030 and 2060 are based upon four socio-economic and environmental scenarios formulated by the Foresight Project [38, 39] and involve combining the spatial assessment of present coastal population with UN statistical demographic data sets (see also Fig. 1 and Table 1). Fundamental to our calculations are the following three assumptions: (i) coastal migration leads to higher relative growth of coastal areas as compared to the landlocked hinterland, (ii) urban and non-urban populations in the coastal zone develop differently and (iii) coastal urban growth is expansive, i.e. urban areas are expanding into previous non-urban space. In order to differentiate coastal from inland growth as well as urban from non-urban growth, we applied correction factors to the respective national growth rates.

**Table 1:** Details on the implemented socio-economic scenarios A-D including population growth variants and coastal correction factors (a, b).

Scenario	Population growth variants	Correction factors		Scenario narratives and assumptions
		Urban (a)	Non-urban (b)	
**Scenario A – Population growth AT HIGH END OF FORECASTS:** High global growth; exclusive social, political and economic governance				
Richer economies	10^th^ perc. ► LOW	1.7	2.0	Fast growing economy and aging population; high demand for low skilled workers including migrants from developing world to regional economic growth poles; declining population growth rates.
Developing world	90^th^ perc. ► HIGH	1.7	2.0	Internal migration in lagging developing countries due to gradual relocation of poverty, rapid migration in faster developing countries.
**Scenario B – Population growth AT LOWEST END OF FORECASTS:** High global growth; inclusive social, political and economic governance				
Richer economies	10^th^ perc. ► LOW	1.7	2.0	High global growth limits overall population growth; very fast ageing population in richer economies; increasing demand for labour but largely voluntary migration from poorer economies.
Developing world	10^th^ perc. ► LOW	2.0	2.0	Relatively equal distribution of growth in economic activity across the world, implying substantial job creation in the urban areas of the poorer economies; massive migration to regional growth poles.
**Scenario C – Population growth TOWARDS HIGHEST END OF FORECASTS:** Low global growth; exclusive social, political and economic governance				
Richer economies	50^th^ perc. ► MEDIUM	1.7	1.7	Stagnant world economic growth; relatively fast aging population; more migration of skilled population from poorer countries; coastal non-urban growth lower compared to the other scenarios, due to stagnant economy and migration to regional growth poles.
Developing world	90^th^ perc. ► HIGH	1.8	1.7	Continuing young population in the poorest parts of the world; stagnant economy and migration to regional growth poles; in general limited internal migration opportunities with more rapid internal migration only in a few faster growing developing countries.
**Scenario D – Population growth AT LOW END OF FORECASTS:** Low global growth; inclusive social, political and economic governance				
Richer economies	50^th^ perc. ► MEDIUM	1.7	2.0	Slow world economic growth; limited demand for labour; low wage growth; aging population; lower levels of migration but rising demand for migrants.
Developing world	50^th^ perc. ► MEDIUM	1.7	2.0	Increased local opportunities for skilled workers in poorer economies; high internal migration in a few faster growing developing countries.

**Scenarios and scenario narratives and assumptions** are based on the Foresight Project’s scenario narratives [38, 39]. Scenarios B and D assume “inclusive governance”, in contrast to “exclusive governance” (scenarios A and C). Inclusive governance e.g. respects human rights, is driven by participatory politics and includes migrant and minority groups in governance structures, while inequalities and tensions between communities determines “exclusive governance” [39].

**Population growth variants**: This column explains the implementation of the Foresight Project’s demographic variants (10^th^ percentile, 50^th^ percentile, 90^th^ percentile) through UN variant of population growth (‘LOW’, ‘MEDIUM’, ‘HIGH’) as provided by the UN’s demographic data sets [48]. Classified as ‘richer economies’, or ‘more developed regions’ in UN terms [46, 47], are Europe, Northern America, Australia/New Zealand (Oceania) and Japan.

**Abbreviations**: perc. = percentile

In total, 187 coastal nations were assessed in this study. It must be noted that Taiwan is not in the UN demographic data sets we employed to build the population projections, so we excluded Taiwan.

### Land area and population in the LECZ

#### Analysis of land area and population in the year 2000

For estimating land and population in the LECZ for the year 2000, we employed the methods of McGranahan et al. [5] and Lichter et al. [6], using an eight-sided connectivity rule to identify the inundation areas that are hydrologically connected to the ocean from the SRTM30 Enhanced Global Map data (Table 2). To differentiate between urban and non-urban population we used the MODIS 500-m Map of Global Urban Extent [42] as proxy for urban areas. For the MODIS urban extent grid, Schneider et al. [42, 43] defined urban areas as „places dominated by built environments“, where the „…‘built environment’ includes all non-vegetative, human-constructed elements, such as roads, buildings, runways, etc. (i.e. human-made surfaces) and ‘dominated’ implies coverage greater than 50% of a given landscape unit (the pixel)” (see Uncertainties, limitations and evaluation of results). For our work we opted for the MODIS 500-m urban map because it provides a more recent and more detailed approximation of urban, built-up and settled areas [42, 43], whereas, for example, the GRUMP urban extent grid [45] has been reported to overestimate urban areas [7, 43]. The MODIS urban extent grid captures most areas of high population density from the GRUMP population data set [44] which we utilised to estimate the baseline population in the LECZ (see Table 2). Consequently, the urban population estimates we produced for the baseline year 2000 represent people living in dense urban areas, while the category of non-urban population summarizes people living in rural areas and those in less densely populated suburban or peri-urban areas. In this aspect, our approach differs from the studies of McGranahan et al. [5] and Balk et al. [1] which used the GRUMP urban extent grids as a base layer for mapping the urban footprint.

**Table 2:** Metrics and data employed for the LECZ and 100-year flood plain baseline assessments (year 2000).

Metrics	Base data
Land area and total population in the LECZ and for 1 m elevation increments within the LECZ; urban population in the LECZ	SRTM30 Enhanced Global Map [80] 30 arc sec resolution
	GTOPO30 Global Digital Elevation Model [82], 30 arc sec (for Greenland)
	Population Count Grid, GRUMP, Alpha Version [44], 30 arc sec, re-sampled to 15 arc sec for analysis of urban/non-urban to match the MODIS data resolution (see below): population year 2000
	Land and Geographic Unit Area Grid, GRUMP, Alpha Version [71], 30 arc sec
	Land and Geographic Unit Area Grid, GPWv3 [83], 2.5 minutes, re-sampled to 30 arc sec (for Greenland)
	MODIS 500-m Map of Global Urban Extent [42, 43], 15 arc sec resolution; population year 2009. Available from: http://www.sage.wisc.edu/people/schneider/research/data.html (accessed June 2011)
	National Administrative Boundaries, GPWv3 [81]
	National Administrative Boundaries, Global Administrative Areas GADM, Level 01 [72] (for Greenland)
	NUTS0 national administrative boundaries [82] (for the Netherlands)
People in the 100-year flood plain	Area extent and total population for 1 m elevation increments within the LECZ (see above)
	National Administrative Boundaries, Global Administrative Areas GADM[72]

We used countries as reporting units (for administrative boundaries see Table 2) and matched the country definitions with the UN classifications [46, 47]. This allowed us to link the spatial population assessments with the population database (see Future LECZ population projections in the years 2030 and 2060). If LECZ population counts and the UN national estimates deviated, which was mostly the case for small island states, corrections were applied adjusting the LECZ counts to match the UN urbanisation and national population data. This procedure ensured consistency between the data sets and the projected LECZ population numbers not exceeding the UN projection totals for the respective countries.

#### Future LECZ population projections in the years 2030 and 2060

Our methodology for projecting the urban and non-urban LECZ population in 2030 and 2060 encompassed two steps. First, UN population estimates and projections per country were developed for each of the Foresight scenarios A–D (Fig. 1) on the basis of the demographic descriptors given in the Foresight Project’s scenario narratives [38, 39]. We matched the latest national low-, medium- and high-population projections of the United Nations’ 2010 Revision of their World Population Prospects [48] to the Foresight scenario assumptions of lower, median and high-end growth predictions (Fig. 1, Table 1 and Table 3). ‘Richer economies’, as stated in the Foresight scenario narratives, were translated to correspond with ‘more developed regions’ as classified by the UN (Japan; Europe; North America; Australia/New Zealand), while countries of the ‘developing world’ (Foresight) were interpreted to belong to the UN’s ‘less developed regions’ (Africa; Asia except for Japan; Latin America and the Caribbean; Oceania except for Australia/New Zealand) [46, 49]. Based on this interpretation, we computed the total future population for all four scenarios A-D and the years 2030 and 2060 per country. Total population was then split into urban and non-urban on the basis of the United Nations’ 2009 Revision of the World Urbanization Prospects [50, 51] and the 2045–2050 trends were used to extrapolate urban and non-urban populations from the latest projection date of the UN urbanisation database (2050) to 2060. Finally, we derived total annual rates of urban (G_ut_) and non-urban (G_nt_) population growth per country from the population data for the periods 2000–2030 and 2030–2060, employing exponential growth functions as described in Balk et al. [52] and Gaffin et al. [53].

**Table 3:** Metrics and data employed for the LECZ and flood plain scenario analyses.

Metrics	Base data
Population in the LECZ projected to 2030 and 2060	Foresight scenario narratives: Scenario narratives and demographic factors [38, 39] (see Fig. 1 and Table 1)
	Total and urban population in the LECZ in 2000 per country (see Table 2)
	World Population Prospects: The 2010 Revision. Total population (both sexes combined) by major area, region and country, annually for 1950–2100 (thousands) [48]
	World Population Prospects: The 2010 Revision. Location list with codes, description, major area, region and development group [47]
	World Urbanization Prospects: The 2009 Revision. Urban Population by Major Area, Region and Country, 1950–2050 [50]
	World Urbanization Prospects: The 2009 Revision. Rural Population by Major Area, Region and Country, 1950–2050 [51]
People in the 100-year flood plain projected to 2030 and 2060	Foresight scenario narratives on sea-level rise 2030: + 10 cm; 2060: + 21 cm [38, 39]
	DIVA 1-in-100-Year Surge Heights [56, 57]
	Total population (year 2000) in the 100-year coastal flood plain in 2000, 2030 and 2060; results per country (see Table 2)
	Coastal population growth rates, country-by-country (intermediate results of LECZ population projections, see above for input data)

In a second step, we projected the urban and non-urban population counts of the LECZ (see Analysis of land area and population in the year 2000) from the reference year 2000 to the years 2030 and 2060 for all scenarios using specific annual rates of coastal urban (G_uc_) and non-urban (G_nc_) population growth of the respective base year (2000, 2030). These growth rates were based on correction factors (a, b) which we developed to account for faster coastal growth as compared to inland growth and on the derived total rates of urban (G_ut_) and non-urban (G_nt_) population growth Equation 1 and Equation 2. This allowed us to differentiate between coastal (G_uc_, G_nc_) and inland (G_ui_, G_ni_) urban and non-urban growth, while controlling the total population growth.

Thus, the **coastal urban growth rate (G**_**uc**_) is given as a function of inland urban growth and the correction factor (a):

$$G_{uc}=a\times G_{ui};\;if\;G_{ui}<0\;then\;G_{uc}=0.001$$ Equation 1

G_uc_ = coastal urban growth rate for the chosen period, e g. 2000–2030;

a = correction factor for coastal urban growth;

G_ui_ = inland urban growth rate for the chosen period, e.g. 2000–2030.

The total urban growth (G_ut_) rate is given as a function of the inland urban growth rate (G_ui_) and the coastal urban growth rate (G_uc_). Both G_ui_ and G_uc_ are weighted by the proportion of the respective population groups (P_ui_; P_uc_) to the total national urban population (P_ut_):

$$G_{ut}=G_{ui}\times \left(P_{ui}÷P_{ut}\right)+G_{uc}\times \left(P_{uc}÷P_{ut}\right)$$ Equation 2

G_ut_ = total urban population growth rate for a period, e.g. 2000–2030;

P_ui_ = inland urban population numbers at beginning of the period;

P_ut_ = total urban population numbers at beginning of the period;

P_uc_ = coastal urban population numbers at beginning of the period.

The coastal urban growth rates (G_uc_) were then derived by solving Equation 2 for G_ui_ and replacing G_ui_ in Equation 1. This step ensures that the aggregate population growth of a country does not exceed the national UN population estimates. The same equations were used for deriving **coastal non-urban population growth rates (G**_**nc**_) from total non-urban population growth rates (G_nt_) and calculating the correction factor for coastal non-urban growth (b).

We also assumed population growth not to decline in the LECZ, even if inland population growth were to be negative. If negative growth occurred, we set G_uc_ = 0.001 and G_nc_ = 0, which generally results in very low growth for coastal urban areas and zero growth for coastal non-urban areas. This procedure was applied for small island states and other countries for which the underlying UN data sets assume negative national growth, such as the Republic of Moldova, Bulgaria, Ukraine, Georgia, Lithuania and Dominica.

The **correction factors for coastal urban and non-urban growth (a, b)** (Table 1) were developed on the basis of the Foresight scenario characteristics regarding economic and societal development, population growth and coastal migration [38, 39], as well as on literature review [11, 13] and expert judgement. They account for the three basic assumptions stated above. We set scenario-specific values for these factors that ranged between 1.7 and 2.0, following earlier studies of Nicholls [13] and Nicholls et al. [11]. Urban expansion leads to an increase in population density, to an expansion of built-up areas into non-urban land through suburbanisation and increasingly to peri-urbanisation effects which creates transient boundaries between urban and non-urban zones [54, 55]. Due to methodological, data- and scale-related constraints, modelling the spatial dynamics linked to these aspects of urban growth was not feasible within the scope of this study. We therefore employed a non-spatial approach to compensate for this limitation: By setting the basic correction factors for coastal non-urban growth (b) higher than the ones for coastal urban growth (a), we accounted for urban expansion by allocating a proportion of the coastal urban growth into the non-urban hinterland (see Equation 1 and Table 1).

According to the assumptions on population growth and migration patterns made in the Foresight Project’s scenario narratives, we set the correction factors (a, b) as follows (see Table 1): Correction factors of 1.7 and 2.0 (for urban and non-urban growth respectively) were applied for scenarios A (population growth at the high end of forecasts) and D (population growth at the low end of forecasts), both for richer economies and for developing countries. Variations were made for scenario B, where we assumed that both coastal urban and coastal non-urban areas in the developing world will be growing at twice the rate of the hinterland. Though ranging at the lowest end of the population forecasts, resulting in stagnation in growth after 2050, the scenario narratives for scenario B outline substantial job creation in urban areas of the poorer economies and massive migration to regional growth poles, which we assume to include coastal urban areas. For scenario C, we adjusted both the coastal urban and the coastal non-urban correction factors as follows: Stagnant economies and migration to regional growth poles were assumed to reduce coastal non-urban growth in comparison to the other scenarios, which is reflected in a lower correction factor (1.7). At the same time, the correction factor for coastal urban growth in the developing world was set slightly higher (1.8) to express the fact that in this scenario internal migration to coastal urban areas is more rapid in some faster growing countries. For richer economies, we see no change for urban areas in comparison to other scenarios.

It must be noted that the underlying UN data, from which we derived the basic national urban and rural growth rates, already consider differences in urban and non-urban (i.e. rural) growth trends and reflect national trends of urbanisation. Our coastal correction factors (a, b) were applied additionally to the derived rates to account for the assumptions that coastal population growth is higher than national population growth in general and that there is urban expansion from 2000 to 2060 into what has been categorised as non-urban areas in the year 2000. Further, we applied the population projections to the LECZ baseline population estimates (year 2000); we did not consider any displacement of the LECZ from sea-level rise and inundation or coastal erosion.

#### People in the 100-year flood plain

The number of people living in the 100-year flood plain was assessed through a slightly modified approach. This was due to data processing constraints in developing spatial representations of the flood plain at a global scale (see Table 2 and Table 3 for base data and metrics). First, we retrieved estimates of the 1-in-100-year extreme water levels from the Dynamic and Interactive Vulnerability Assessment (DIVA) database [56, 57] (Table 2). From these we computed the average 1-in-100-year surge height per level-1 administrative unit (3,366 units in total). Several small coastal countries and island states (i.e. Anguilla, Maldives and Singapore) had no records in the GADM Level-01 data set. For these we employed the GADM Level 0 data set and averaged the storm surge heights per country. The derived average storm surge heights were then displaced upwards by the amount of global mean sea-level rise assumed for the 2030 and 2060 Foresight scenarios [38, 39], 10 cm and 21 cm respectively (Table 3). It must be noted that the actual sea-level rise may vary considerably between regions and scenarios beyond the 2030/2060 narratives [23, 24]. Also, the analysis does not consider possible future climate-induced changes in storm or cyclone activity and resulting effects on flood levels.

We calculated the population in the flood plain based on the distribution of coastal population per 1 m elevation increment (Table 2) assuming that all land below the computed surge heights belongs to the 100-year flood plain. To account for the limited vertical resolution of the employed SRTM30 digital elevation model (multiples of 1 m), we assumed that population distribution within elevation increments is homogeneous. In order to account for differences in the land-ocean boundaries of the employed datasets, we allocated GRUMP population pixels that were falling in the ocean to the nearest GADM administrative units. The derived flood plain population represents the baseline (year 2000) population within the 2000, 2030 and 2060 flood plain. Next, these population estimates were projected into 2030 and 2060 by applying the LECZ’s total coastal growth per country. Since the flood plain could not be defined spatially in this study with the methods applied, differentiating between urban and non-urban flood plain population was not possible.

### Results

**Fig 2:**
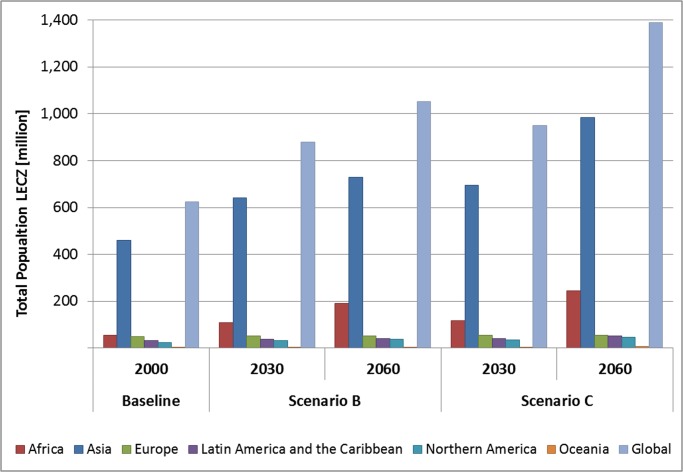
LECZ population in the year 2000 and projections for 2030/2060 per continent, scenarios A-D.

**Fig 3:**
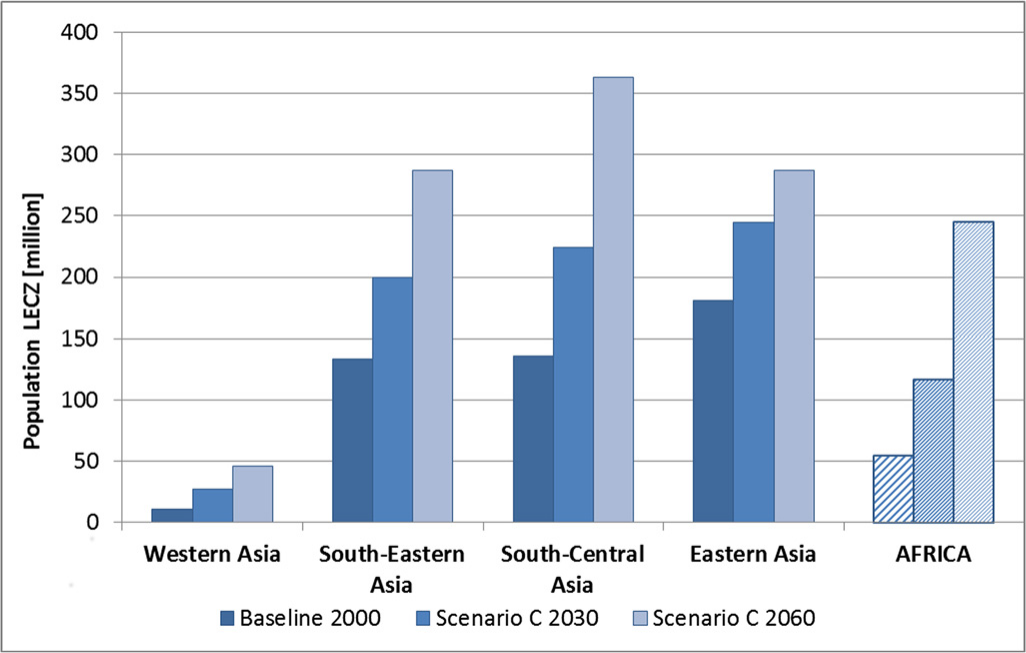
LECZ population in Asia in the year 2000 and projections for 2030/2060 per region, scenario C. Included are totals of LECZ population in Africa for the baseline year 2000 and for 2030/2060.

**Fig 4:**
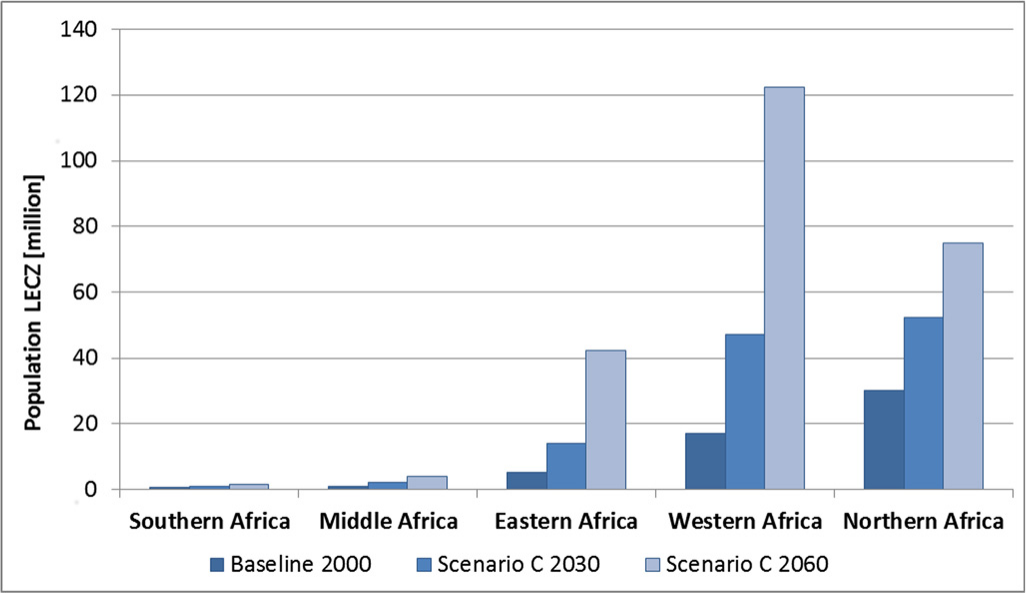
LECZ population in Africa in the year 2000 and projections for 2030/29160 per region, scenario C.

In the following sections, we present the results of our assessments at aggregated continental and regional scales (see Table 4, Table 5 and Table 8; Fig. 2, Fig. 3 and Fig. 4; S1 Table, S2 Table and S3 Table), as well as country-specific results of the top 25 countries in terms of population exposure (Table 6 and Table 7). We focus on two of the four Foresight scenarios assessed, unless the results require further attention: Scenario B (population growth at the lowest end of forecasts) and scenario C (population growth towards the highest end of forecasts). As supporting information, S4 Table lists all assessment results as well as the demographic input data per reporting unit, i.e. per country.

**Table 4:** LECZ population in the year 2000 and projections for 2030/2060 per continent and development status, scenarios A-D.

Region	LECZ population in 2000			LECZ population in 2030				LECZ population in 2060			
	Baseline 2000 [million]	Urban [%]	Non-urban [%]	Scenario A [million]	Scenario B [million]	Scenario C [million]	Scenario D [million]	Scenario A [million]	Scenario B [million]	Scenario C [million]	Scenario D [million]
**World**	**625.2**	**23.5**	**76.5**	**938.9**	**879.1**	**948.9**	**892.9**	**1,318.3**	**1,052.8**	**1,388.2**	**1,128.1**
More dev. regions	107.5	50.1	49.9	120.6	120.6	125.8	125.9	124.1	124.1	138.4	138.4
Less dev. regions and least dev. countries	517.7	18.0	82.0	818.4	758.6	823.1	767.1	1,194.1	928.6	1,249.8	989.7
Least dev. countries	93.0	7.1	92.9	146.9	132.5	146.5	136.3	231.4	181.9	242.0	192.7
Less dev. regions, excluding least dev. countries	424.7	20.4	79.6	671.5	626.1	676.6	630.7	962.8	746.7	1,007.7	797.0
Less dev. regions, excluding China	373.7	17.9	82.1	619.3	561.4	619.0	574.6	958.8	729.1	1,005.0	785.5
China	144.0	18.1	81.9	199.0	197.2	204.1	192.4	235.4	199.6	244.8	204.2
Sub-Saharan Africa	24.2	17.8	82.2	66.4	63.1	65.7	61.3	160.0	136.5	174.0	126.6
**AFRICA**	54.2	16.5	83.5	117.6	108.5	116.8	108.9	229.3	190.0	245.2	185.6
**ASIA**	460.8	20.1	79.9	688.7	640.3	695.0	649.4	943.9	728.6	983.3	792.8
**EUROPE**	50.0	40.2	59.8	52.8	52.8	54.5	54.5	52.1	52.1	55.7	55.7
**LATIN AMERICA AND THE CARIBBEAN**	32.2	28.8	71.2	41.7	39.5	42.3	39.8	50.6	40.1	52.3	42.6
**NORTHERN AMERICA**	24.6	59.6	40.4	33.5	33.5	35.5	35.5	37.0	37.0	45.5	45.5
**OCEANIA**	3.3	34.7	65.3	4.7	4.6	4.8	4.8	5.5	5.0	6.1	5.8

Classifications by major region and develoment status follow the UN classification scheme [46, 47]. **Abbreviations**: dev. = developed.

**Table 5:** Population projections for the LECZ and the 100-year flood plain for 2030/2060 per continent, scenarios A-D.

Region	Baseline population			Scenario	Total population		LECZ population				People in the 100-year flood plain					
	Total 2000 [million]	LECZ 2000 [million]	Flood plain 2000 [million]	Results per scenario and year	2030 [million]	2060 [million]	2030 [million]	% of total pop. 2030	2060 [million]	% of total pop. 2060	2030 [million]	% of total pop. 2030	% of LECZ pop. 2030	2060 [million]	% of total pop. 2060	% of LECZ pop. 2060
**World**	**6,100.8**	**625.2**	**189.2**	**A**	**8,625.1**	**11,064.2**	**938.9**	**100.0**	**1,318.3**	**100.0**	**282.2**	**100.0**	**30.1**	**392.9**	**100.0**	**29.8**
				**B**	**7,845.7**	**7,925.1**	**879.1**	**100.0**	**1,052.8**	**100.0**	**268.1**	**100.0**	**30.5**	**315.5**	**100.0**	**30.0**
				**C**	**8,688.7**	**11,279.4**	**948.9**	**100.0**	**1,388.2**	**100.0**	**285.9**	**100.0**	**30.1**	**411.3**	**100.0**	**29.6**
				**D**	**8,298.5**	**9,597.0**	**892.9**	**100.0**	**1,128.1**	**100.0**	**271.0**	**100.0**	**30.4**	**339.5**	**100.0**	**30.1**
AFRICA	811.1	54.2	12.6	A	1,641.4	2,955.3	117.6	12.5	229.3	17.4	26.0	9.2	22.1	46.9	11.9	20.5
				B	1,482.8	2,115.1	108.5	12.3	190.0	18.0	23.5	8.8	21.7	37.9	12.0	20.0
				C	1,641.4	2,955.3	116.8	12.3	245.2	17.7	25.7	9.0	22.0	49.2	12.0	20.1
				D	1,562.0	2,512.2	108.9	12.2	185.6	16.5	24.1	8.9	22.1	38.4	11.3	20.7
ASIA	3,697.1	460.8	137.3	A	5,107.7	6,153.9	688.7	73.4	943.9	71.6	211.1	74.8	30.6	297.6	75.7	31.5
				B	4,571.9	4,174.5	640.3	72.8	728.6	69.2	199.9	74.6	31.2	231.6	73.4	31.8
				C	5,113.0	6,170.3	695.0	73.2	983.3	70.8	213.4	74.7	30.7	309.6	75.3	31.5
				D	4,844.8	5,104.5	649.4	72.7	792.8	70.3	200.7	74.0	30.9	250.7	73.8	31.6
EUROPE	726.8	50.0	28.2	A	704.3	582.5	52.8	5.6	52.1	3.9	30.1	10.7	57.0	30.2	7.7	57.9
				B	704.3	582.5	52.8	6.0	52.1	4.9	30.1	11.2	57.0	30.2	9.6	57.9
				C	741.2	702.3	54.5	5.7	55.7	4.0	31.2	10.9	57.2	32.4	7.9	58.1
				D	741.2	702.3	54.5	6.1	55.7	4.9	31.2	11.5	57.2	32.4	9.5	58.0
LATIN AMERICA AND THE CARIBBEAN	521.4	32.2	6.1	A	743.5	923.0	41.7	4.4	50.6	3.8	8.1	2.9	19.5	10.2	2.6	20.2
				B	660.0	610.6	39.5	4.5	40.1	3.8	7.7	2.9	19.4	7.9	2.5	19.8
				C	743.5	923.0	42.3	4.5	52.3	3.8	8.2	2.9	19.5	10.5	2.6	20.2
				D	701.6	753.2	39.8	4.5	42.6	3.8	7.7	2.9	19.4	8.5	2.5	20.0
NORTHERN AMERICA	313.3	24.6	4.2	A	381.9	393.4	33.5	3.6	37.0	2.8	5.8	2.0	17.2	6.5	1.7	17.7
				B	381.9	393.4	33.5	3.8	37.0	3.5	5.8	2.2	17.2	6.5	2.1	17.7
				C	401.7	466.3	35.5	3.7	45.5	3.3	6.1	2.1	17.2	8.0	1.9	17.5
				D	401.7	466.3	35.5	4.0	45.5	4.0	6.1	2.3	17.2	8.0	2.4	17.5
OCEANIA	31.1	3.3	0.8	A	46.2	56.2	4.7	0.5	5.5	0.4	1.2	0.4	25.6	1.5	0.4	27.2
				B	44.7	49.1	4.6	0.5	5.0	0.5	1.2	0.4	25.2	1.3	0.4	25.9
				C	47.8	62.1	4.8	0.5	6.1	0.4	1.2	0.4	25.3	1.6	0.4	26.5
				D	47.1	58.4	4.8	0.5	5.8	0.5	1.2	0.4	25.2	1.5	0.4	25.8

**Total population** is based on [47, 48]. Classifications by major region and develoment status follow the UN classification scheme [46, 47]. All LECZ areas and population numbers are based on own assessments. **Abbreviations**: pop. = population.

**Table 6:** Top 25 coastal countries with highest LECZ population in the year 2000, ranked by LECZ population.

Rank LECZ 2000	Country	Region	Development status	Land area and population per country in 2000					LECZ area and population per country in 2000							
				Total land area [km^2^]	Total pop. [million]	Urban pop. [%]	Non-urban pop. [%]	Pop. density [p/km^2^]	LECZ area [km^2^]	LECZ area in % of total area	LECZ pop. [million]	Urban pop. [%]	Non-urban pop. [%]	Pop. density [p/km^2^]	LECZ pop. in % of total pop.	LECZ pop. in % of global LECZ pop.
**1**	China	Eastern Asia	less dev.	9,197,930	1,269.1	35.8	64.2	138	181,909	2.0	**144.0**	18.1	81.9	792	11.3	23.03
**2**	India	S-Central Asia	less dev.	3,211,220	1,053.9	27.7	72.3	328	82,262	2.6	**63.9**	16.4	83.6	777	6.1	10.23
**3**	Bangladesh	S-Central Asia	least dev.	135,986	129.6	23.6	76.4	953	54,679	40.2	**63.1**	4.4	95.6	1,154	48.7	10.10
**4**	Viet Nam	S-Eastern Asia	less dev.	328,594	78.8	24.5	75.5	240	66,232	20.2	**43.1**	12.9	87.1	650	54.7	6.89
**5**	Indonesia	S-Eastern Asia	less dev.	1,901,200	213.4	42.0	58.0	112	172,092	9.1	**39.3**	29.7	70.3	228	18.4	6.28
**6**	Japan	Eastern Asia	more dev.	372,304	125.7	65.2	34.8	338	24,154	6.5	**30.2**	59.7	40.3	1,250	24.0	4.83
**7**	Egypt	Northern Africa	less dev.	969,160	67.6	42.8	57.2	70	23,676	2.4	**25.5**	14.7	85.3	1,075	37.6	4.07
**8**	U.S.	North. America	more dev.	9,130,850	282.5	79.1	20.9	31	235,336	2.6	**23.4**	61.4	38.6	99	8.3	3.74
**9**	Thailand	S-Eastern Asia	less dev.	516,525	63.2	31.1	68.9	122	35,375	6.8	**16.4**	38.2	61.8	464	26.0	2.63
**10**	Philippines	S-Eastern Asia	less dev.	295,298	77.3	48.0	52.0	262	20,165	6.8	**13.0**	11.9	88.1	643	16.8	2.07
**11**	Myanmar	S-Eastern Asia	least dev.	669,464	45.0	27.8	72.2	67	48,651	7.3	**12.5**	11.4	88.6	257	27.8	2.00
**12**	Brazil	S America	less dev.	8,485,010	174.4	81.2	18.8	21	121,668	1.4	**11.6**	34.7	65.3	95	6.6	1.85
**13**	Netherlands	Western Europe	more dev.	35,376	15.9	76.8	23.2	448	24,870	70.3	**11.6**	68.1	31.9	464	72.8	1.85
**14**	Nigeria	Western Africa	less dev.	904,537	123.7	42.5	57.5	137	14,973	1.7	**7.4**	16.0	84.0	491	5.9	1.18
**15**	United Kingdom	Northern Europe	more dev.	247,576	58.9	78.7	21.3	238	21,389	8.6	**7.1**	30.6	69.4	331	12.0	1.13
**16**	Mexico	Central America	less dev.	1,942,800	100.0	74.7	25.3	51	93,369	4.8	**5.6**	7.5	92.5	60	5.6	0.90
**17**	Italy	Southern Europe	more dev.	299,309	57.0	67.2	32.8	190	18,794	6.3	**5.4**	26.8	73.2	286	9.4	0.86
**18**	Malaysia	S-Eastern Asia	less dev.	330,615	23.4	62.0	38.0	71	19,506	5.9	**5.2**	17.4	82.6	265	22.1	0.83
**19**	Germany	Western Europe	more dev.	356,104	82.3	73.1	26.9	231	22,600	6.3	**4.6**	24.1	75.9	203	5.6	0.73
**20**	Pakistan	S-Central Asia	less dev.	785,150	144.5	33.1	66.9	184	22,835	2.9	**4.6**	38.0	62.0	200	3.2	0.73
**21**	Argentina	South America	less dev.	2,736,850	36.9	90.1	9.9	13	52,026	1.9	**3.8**	58.0	42.0	73	10.3	0.61
**22**	Russian Fed.	Eastern Europe	more dev.	16,677,500	146.8	73.3	26.7	9	271,546	1.6	**3.5**	16.1	83.9	13	2.4	0.56
**23**	Cambodia	S-Eastern Asia	least dev.	179,416	12.4	16.9	83.1	69	13,493	7.5	**3.2**	2.5	97.5	237	25.7	0.51
**24**	Spain	South. Europe	more dev.	505,164	40.3	76.3	23.7	80	6,286	1.2	**3.2**	35.5	64.5	505	7.9	0.51
**25**	Rep. of Korea	Eastern Asia	less dev.	99,006	46.0	79.6	20.4	464	4,572	4.6	**3.0**	33.6	66.4	654	6.5	0.48

**Total land area** was calculated through zonal statistics using the following data sets: land area grids from GRUMP Alpha and GPWv3 (for Greenland), national boundaries from GPWv3, NUTS0 (Netherlands) and Global Administrative Areas (Greenland) (see Table 2). **Total population** including urban/non-urban shares is based on [47, 48, 50, 51]. Classifications by major region and develoment status follow the UN classification scheme [46, 47]. All LECZ areas and population numbers are based on own assessments. **Abbreviations**: U.S. = United States of America; Russian Fed. = Russian Federation; Rep. of Korea = Republic of Korea; S. = South; dev. = developed; pop. = population.

**Table 7:** Top 25 countries with highest LECZ population and people in the 100-year flood plain in 2030/2060, ranked by LECZ Scenario C 2060.

Rank LECZ Scenario C 2060	Rank LECZ 2000	Country	Total population			LECZ population				People in the 100-year flood plain			
			Baseline 2000 [million]	Scenario C 2030 [million]	Scenario C 2060 [million]	Baseline 2000 [million]	Scenario C 2030 [million]	Scenario C 2060 [million]	Pop. growth 2000–2060 [%]	Baseline 2000 [million]	Scenario C 2030 [million]	Scenario C 2060 [million]	Pop. growth 2000–2060 [%]
**1**	1	China	1,269.1	1,467.4	1,467.7	144.0	204.1	**244.8**	170	56.0	82.8	103.4	185
**2**	2	India	1,053.9	1,612.0	2,096.0	63.9	120.8	**216.4**	339	17.1	33.8	63.6	372
**3**	3	Bangladesh	129.6	193.5	237.7	63.1	85.1	**109.5**	173	6.0	8.8	12.4	207
**4**	5	Indonesia	213.4	296.5	354.3	39.3	61.9	**93.7**	239	5.4	9.1	14.5	267
**5**	4	Viet Nam	78.8	107.6	123.1	43.1	58.7	**80.4**	187	26.3	36.4	50.6	192
**6**	7	Egypt	67.6	112.7	154.6	25.5	45.0	**63.5**	249	7.4	13.8	20.7	281
**7**	14	Nigeria	123.7	269.3	534.3	7.4	19.8	**57.7**	785	0.1	0.3	0.9	839
**8**	8	U.S.	282.5	361.7	421.0	23.4	34.0	**43.9**	188	3.5	5.3	7.1	200
**9**	9	Thailand	63.2	77.4	83.0	16.4	24.7	**36.8**	224	3.5	5.6	9.1	262
**10**	10	Philippines	77.3	133.7	199.8	13.0	23.8	**34.9**	270	2.0	3.8	5.8	293
**11**	6	Japan	125.7	120.2	103.2	30.2	32.1	**32.7**	108	8.3	9.2	9.7	117
**12**	20	Pakistan	144.5	247.8	341.8	4.6	12.7	**30.1**	660	0.7	2.2	5.7	782
**13**	11	Myanmar	45.0	57.7	66.2	12.5	16.4	**22.8**	182	3.1	4.3	6.3	206
**14**	27	Senegal	9.5	21.0	38.7	2.9	8.5	**19.2**	655	0.4	1.1	2.7	761
**15**	12	Brazil	174.4	233.9	268.3	11.6	15.8	**18.7**	162	2.1	2.9	3.5	168
**16**	29	Iraq	23.9	58.0	114.5	2.7	9.3	**18.1**	679	1.3	4.7	9.3	708
**17**	40	Benin	6.5	15.3	29.6	1.4	5.4	**15.0**	1,058	0.1	0.6	1.6	1,121
**18**	57	Un. Rep. of Tanzania	34.0	85.7	200.7	0.6	2.8	**14.0**	2,203	0.2	0.9	4.3	2,302
**19**	13	Netherlands	15.9	17.3	17.0	11.6	12.3	**11.8**	103	9.5	10.2	9.8	103
**20**	18	Malaysia	23.4	39.3	54.2	5.2	7.8	**11.3**	218	0.4	0.7	1.1	243
**21**	64	Somalia	7.4	17.0	40.9	0.6	2.2	**9.8**	1,683	0.2	0.6	2.7	1,695
**22**	15	United Kingdom	58.9	69.3	73.5	7.1	8.0	**8.8**	124	3.8	4.4	4.8	128
**23**	47	Côte d'Ivoire	16.6	31.4	54.0	1.2	3.0	**7.6**	644	0.1	0.3	0.7	647
**24**	21	Argentina	36.9	49.4	62.3	3.8	5.6	**7.6**	200	0.9	1.4	2.0	212
**25**	31	Mozambique	18.2	37.8	68.1	2.3	4.4	**7.5**	325	0.7	1.4	2.5	360
29	17	Italy	5.4	5.9	6.1	5.4	5.9	**6.1**	112	2.1	2.4	2.7	128
30	23	Cambodia	12.4	18.5	23.4	3.2	4.7	**6.0**	187	0.6	1.0	1.4	224
35	19	Germany	82.3	79.5	72.4	4.6	4.7	**4.7**	101	3.1	3.2	3.2	103
37	24	Spain	40.3	50.0	49.9	3.2	3.9	**4.1**	129	1.3	1.6	1.7	134
42	25	Republic of Korea	46.0	52.9	52.8	3.0	3.5	**3.6**	122	1.3	1.5	1.6	128
44	22	Russian Federation	146.8	136.4	120.8	3.5	3.5	**3.5**	101	1.4	1.4	1.4	104

**Total population** per country was based on [47, 48]. All LECZ areas and population numbers are based on own assessments (see Material and Methods, Table 2 and Table 3). **Abbreviations**: U.S. = United States of America; Un. Rep. of Tanzania = United Republic of Tanzania; pop. = population

**Table 8:** People in the 100-year flood plain in the year 2000 and projections for 2030/2060 per continent and development status, scenarios A-D.

Region	Total population	LECZ population	People in the 100-year flood plain										
	2000	2000	2000			2030				2060			
	Baseline [million]	Baseline [million]	Baseline [million]	% of LECZ pop.	% of flood plain pop.	Scenario A [million]	Scenario B [million]	Scenario C [million]	Scenario D [million]	Scenario A [million]	Scenario B [million]	Scenario C [million]	Scenario D [million]
**WORLD**	**6,100.8**	**625.2**	**189.2**	**30.3**	**100.0**	**282.2**	**268.1**	**285.9**	**271.0**	**392.9**	**315.5**	**411.3**	**339.5**
More developed regions	1,188.8	107.5	41.2	38.4	21.8	45.4	45.4	47.3	47.3	46.9	46.9	51.0	51.0
Less developed regions and least developed countries	4,912.0	517.7	147.9	28.6	78.2	236.8	222.6	238.6	223.7	346.0	268.6	360.3	288.4
Least developed countries	662.0	93.0	12.6	13.6	6.7	22.9	20.9	22.6	21.3	41.6	33.8	43.8	34.2
Less developed regions, excluding least developed countries	4,250.0	424.7	135.3	31.8	71.5	213.9	201.7	215.9	202.5	304.5	234.8	316.5	254.2
Less developed regions, excluding China	3,642.9	373.7	91.9	24.6	48.6	156.0	142.6	155.7	145.6	246.7	184.4	256.9	202.2
China	1,269.1	144.0	56.0	38.9	29.6	80.8	80.0	82.8	78.1	99.4	84.3	103.4	86.2
Sub-Saharan Africa	669.1	24.2	3.4	14.2	1.8	9.6	9.0	9.4	8.9	23.6	20.0	25.3	18.6
**AFRICA**	811.1	54.2	12.6	23.3	6.7	26.0	23.5	25.7	24.1	46.9	37.9	49.2	38.4
Eastern Africa	251.6	5.2	1.4	28.1	0.8	4.3	4.0	4.0	3.9	11.8	10.4	12.7	9.3
Middle Africa	96.2	1.1	0.2	13.9	0.1	0.3	0.3	0.3	0.3	0.5	0.4	0.5	0.4
Northern Africa	176.2	30.3	9.2	30.3	4.9	16.3	14.6	16.3	15.2	23.3	18.1	24.0	19.9
Southern Africa	51.4	0.5	0.1	21.1	0.1	0.2	0.2	0.2	0.2	0.3	0.2	0.3	0.2
Western Africa	235.7	17.1	1.7	10.1	0.9	4.8	4.5	4.8	4.5	11.0	8.9	11.7	8.7
**ASIA**	3,697.1	460.8	137.3	29.8	72.6	211.1	199.9	213.4	200.7	297.6	231.6	309.6	250.7
Eastern Asia	1,473.3	180.9	67.2	37.1	35.5	93.4	92.3	95.8	90.9	113.3	97.2	117.7	99.9
South-Central Asia	1,515.6	135.7	25.0	18.4	13.2	46.9	40.7	46.3	42.6	79.2	58.2	83.5	63.8
South-Eastern Asia	523.8	133.2	41.4	31.1	21.9	60.3	57.1	61.0	57.4	86.4	61.8	89.0	71.9
Western Asia	184.4	11.1	3.8	34.0	2.0	10.6	9.8	10.3	9.9	18.8	14.4	19.3	15.0
**EUROPE**	726.8	50.0	28.2	56.3	14.9	30.1	30.1	31.2	31.2	30.2	30.2	32.4	32.4
Eastern Europe	304.2	6.8	2.7	39.7	1.4	2.8	2.8	2.8	2.8	2.9	2.9	2.9	2.9
Northern Europe	94.3	11.2	5.1	45.6	2.7	5.6	5.6	5.8	5.8	5.6	5.6	6.2	6.2
Southern Europe	145.1	10.6	4.3	40.2	2.3	5.0	5.0	5.1	5.1	5.3	5.3	5.7	5.7
Western Europe	183.1	21.4	16.1	75.2	8.5	16.7	16.7	17.5	17.5	16.4	16.4	17.6	17.6
**LATIN AMERICA AND THE CARIBBEAN**	521.4	32.2	6.1	18.9	3.2	8.1	7.7	8.2	7.7	10.2	7.9	10.5	8.5
Caribbean	38.4	3.5	0.7	19.1	0.4	0.8	0.8	0.8	0.8	1.0	0.8	1.1	0.9
Central America	135.6	6.8	1.0	14.4	0.5	1.2	1.1	1.2	1.1	1.3	1.2	1.4	1.2
South America	347.4	21.9	4.4	20.2	2.3	6.1	5.8	6.2	5.8	7.8	5.9	8.1	6.4
**NORTHERN AMERICA**	313.3	24.6	4.2	17.1	2.2	5.8	5.8	6.1	6.1	6.5	6.5	8.0	8.0
**OCEANIA**	31.1	3.3	0.8	24.4	0.4	1.2	1.2	1.2	1.2	1.5	1.3	1.6	1.5
Australia/New Zealand	23.0	2.7	0.5	20.5	0.3	0.7	0.7	0.8	0.8	0.8	0.8	1.0	1.0
Melanesia	7.0	0.4	0.1	31.9	0.1	0.3	0.2	0.2	0.2	0.4	0.3	0.4	0.3
Micronesia	0.5	0.2	0.1	55.5	0.1	0.1	0.1	0.1	0.1	0.2	0.1	0.2	0.2
Polynesia	0.6	0.1	0.1	40.2	0.0	0.1	0.1	0.1	0.1	0.1	0.1	0.1	0.1

**Total population** is based on [47, 48]. Classifications by major region and develoment status follow the UN classification scheme [46, 47]. All LECZ areas and population numbers are based on own assessments. **Abbreviations**: pop. = population; flood pl. = flood plain.

### Population in the LECZ in 2000, 2030 and 2060

The **LECZ** comprised only 2.3% (2,599 thousand km^2^) of the total land area of all coastal countries, but 10.9% (625 million) of their population in the year 2000 (Table 4; S1 Table). The majority (83%) of the global LECZ population lived in less developed countries. The average LECZ population density in the year 2000 was 241 people/km^2^, which was more than five times higher than the global mean (47 people/km^2^). The highest average population densities in terms of development status were found in the LECZ of least developed countries (382 people/km^2^). Our results suggest a growth of the population in the LECZ from 625 million (year 2000; global population of 6.1 billion) to between 879 million (scenario B; global population: 7.8 billion) and 949 million people (scenario C; global population: 8.7 billion) in the year 2030 (Table 4 and Table 5; Fig. 2; S3 Table). By 2060, the LECZ population is likely to approach 1.4 billion people (534 people/km^2^) under the highest-end growth assumption, which would be 12% of the world’s population of 11.3 billion (scenario C). Even when assuming population growth at the lowest end of the forecasts (scenario B), we estimate there to be more than one billion people in the LECZ globally by 2060 with an average population density of 405 people/km^2^.

**Asia** had the largest LECZ population in the year 2000 (461 million or 73% of the total LECZ population; Table 4 and Fig. 2; S2 Table), and this will also be the case in 2030 and 2060, under all scenarios. By 2060, between 729 million (scenario B) and 983 million (scenario C) people will be living in the LECZ in Asia, which amounts to around 70% of the world’s LECZ population. Within Asia, **Eastern Asia** (China, Hong Kong Special Administrative Region, Macao Special Administrative Region, Democratic People's Republic of Korea, Republic of Korea, Japan) had the largest proportion of population in the LECZ and showed the highest LECZ population density worldwide in the year 2000 (839 people/km^2^; Fig. 3 and S1 Table). However, the projections suggest that **South–Central Asia** (Bangladesh, India, Islamic Republic of Iran, Maldives, Pakistan, Sri Lanka) will contribute more to the overall coastal population growth than Eastern Asia in the next decades and is projected to have the highest population totals in the LECZ of all Asian regions by 2060 (Fig. 3; S2 Table). This is mainly due to the large populations of Bangladesh, India and Pakistan, in conjunction with significantly higher rates of change as implied in the underlying demographic data sets [48, 50, 51].

Though **China** represented the largest proportion of people in the LECZ in the year 2000 (144 million people, 11.3% of its total population and 23% of the global LECZ population), its population growth is projected to slow down after 2030 (Table 6). Nevertheless, China could still grow to reach between 200 million (scenario B) and 245 million (scenario C; 16.7% of their total population) people in the LECZ by the year 2060, more than any other nation (Table 7; S2 Table). China is closely followed by **India**, which could experience a three-fold increase of its LECZ population between the baseline year 2000 (64 million; 6.1% of its total population) and the year 2060 (216 million; 10.3% of its total population) under the high-growth scenario C (Table 6 and Table 7). The LECZ population of **Bangladesh** (63 million) was similar to India (64 million) in the baseline year 2000 (Table 6). However, the LECZ of Bangladesh comprises over 40% of the country’s total land area (India: 2.6% of the total land area) and had a much larger share of the country’s total population (49%) than India (6.1%) in 2000. Further, the LECZ population was predominantly non-urban (96%) and the population density was considerably higher (1,154 people/km^2^) than the respective of India (777 people/km^2^) in the baseline year. Nevertheless, the projections for Bangladesh under scenario C assume a slower growth for its LECZ population, which can be explained by relatively lower non-urban coastal growth (in comparison to other scenarios) in conjunction with the very large share of non-urban population (see Table 1 and Table 7 and Table 1). **Pakistan**, the third country in South-Central Asia that ranks among the top-25 countries in terms of LECZ population both in the 2000 and in 2060, is projected to encounter the strongest population growth in this region under scenario C (Table 6 and Table 7). In the year 2000, not a very large share of the Pakistani population was located in low-lying coastal areas (3.2% or 4.6 million people). However, the LECZ population could increase six-fold to reach 30 million people by 2060.

**China, India, Bangladesh, Indonesia and Viet Nam** represent the five countries with the largest share of population in the LECZ worldwide (Table 6). All these countries are located in Eastern, South-Central and South-Eastern Asia and belong to the less and least developed nations of the world. Together they accounted for 56% of the global LECZ population in the year 2000 (353 million people; 5.8% of the world population). From these countries, Bangladesh had the highest proportion of people living in low-lying coastal areas (49% of their total population respectively). All countries were characterised by very large extends of non-urban settlements in the LECZ, between 70% (Indonesia) and 96% (Bangladesh). According to our population projections, these countries will maintain the top five positions in the future and count up to 745 million people in the LECZ by 2060, 6.6% of the world population (scenario C; Table 7).

In contrast to Asia, **Africa’s** LECZ population (54 million in 2000, 8.7% of the African coastal countries’ population) and coastal land area in the LECZ (194 thousand km^2^; 0.9% of the African coastal countries land area) are considerably smaller (Table 4, Fig. 2 and Fig. 3; S2 Table). However, Africa will be the continent to experience the highest rates of growth and urbanisation in the LECZ across all scenarios. In particular, the LECZ population of **Sub-Saharan Africa** (all of Africa except Northern Africa; includes the Sudan), which represented 45% of the African nations’ LECZ population in 2000, could grow from 24 million (2000) to 66 million by 2030 and to 174 million by 2060 (both scenario C) due to an average coastal growth rate of up to 3.3% (2000–2030) and 3.2% (2030–2060). These rates are considerably higher than in Asia, where annual rates of growth are expected to reach 1.4% in the first three decades (2000–2030) and afterwards drop to 1.2% (scenario C).

Among the African regions, coastal population growth is projected to be highest in Eastern and Western Africa, especially in the urban centres of Western Africa where between 72 million (scenario B) and 94 million (scenario C) people will reside by 2060 (Fig. 4; S2 Table). **Northern Africa** (Algeria, Egypt, Libya, Morocco, Sudan, Tunisia, Western Sahara) had the largest LECZ population in the year 2000 (30 million), but will not keep pace with the coastal growth in **Western Africa** where nations like **Nigeria, Benin, Côte d'Ivoire and Senegal** are growing considerably faster. According to our projections, all four countries will be among the top-25 countries in terms of LECZ population totals by 2060 (Table 7), while in the baseline year 2000 only Nigeria was present in this top-25 ranking with 58 million people (11% of its population). All of them will experience a considerable population increase. A characteristic example is Senegal, which had a small LECZ population in the year 2000 (2.9 million) and where 50% of the country’s total population could live on low-lying coastal land by 2060 (19 million people; Table 7). In **Eastern Africa**, the countries of **Tanzania, Somalia and Mozambique** boost the regional development through strong coastal growth. These three countries are expected to feature among the top-25 countries with the highest population in the LECZ by the year 2060 (scenario C; Table 7), in stark contrast with their comparatively low LECZ population in 2000 (Table 6 and Table 7). The United Republic of Tanzania is projected to undergo a 22-fold rise in LECZ population numbers and Somalia a 16-fold increase, while Mozambique is expected to triple its LECZ population (all scenario C). **Southern Africa**, which comprises the coastal countries Namibia and South Africa, exhibited the smallest LECZ population with 0.5 million people in the year 2000, increasing to 1.7 million by 2060 (Scenarios C; Fig. 3).

**Egypt** (26 million; 38% of its total population) and **Nigeria** (7.4 million; 5.9% of its total population) were the countries with the highest population in the LECZ in the African continent in 2000, ranking at places 6 and 7 globally (Table 6). The Egyptian LECZ along the Mediterranean coast and the Nile delta (1,075 people/km^2^) was almost as densely populated as the LECZ of Japan (1,250 people/km^2^) or Bangladesh (1,154 people/km^2^) in 2000. However, only 15% of the LECZ population actually lived in dense urban areas in the year 2000. By 2030, population density along the Egyptian coast is expected to increase to 1,902 people/km^2^ and to 2,681 people/km^2^ by 2060.

In **Europe**, the total population in the LECZ (50 million) was similar to that in Africa (54 million) in the year 2000, while the LECZ area was more than double in size (Europe: 471 thousand km^2^; Africa: 194 thousand km^2^; S1 Table). This resulted in an average population density of only 106 people/km^2^ in the in European LECZ, as opposed to the 280 people/km^2^ in the LECZ of Africa or to the global average of 241 people/km^2^. Also, the proportion of urban population in the LECZ in Europe (40%) was significantly higher than in Asia (20%) or Africa (16.5%) in the year 2000 (Table 4). Among the European regions, Western Europe stands out with about 21 million people living in a LECZ that is quite densely populated (328 people/km^2^ respectively), half of which is located in the **Netherlands** (12 million; 73% of its total population). However, the LECZ of Europe, as a region that is characterised by **richer economies,** is projected to experience only low to moderate population growth towards 56 million people by the year 2060, at most (scenario D). In contrast to Europe, Africa could more than quadruple its LECZ population in the same period. From the six European countries with the highest population in the LECZ in the year 2000 (Netherlands, United Kingdom, Italy, Germany, Spain and the Russian Federation), only the Netherlands and the United Kingdom will, according to our projections, rank among the top-25 countries in 2060, though dropping in rank compared to the year 2000 (Table 6 and Table 7). The Russian Federation has the largest LECZ (272 thousand km^2^) of all countries worldwide. In 2000, 3.51 million people (2.4% of the national total; Table 6) were living in the Russian LECZ, but little change is expected here with LECZ population reaching at maximum 3.55 million by 2060 (scenario C). In accordance with the UN’s classification, the Russian Federation is assigned to Eastern Europe [46].

**Northern America** (Bermuda, Canada, Greenland, Saint Pierre and Miquelon, United States of America) has the second largest extent of LECZ after Asia with over 507 thousand km^2^ (see S2 Table). However, the overall number of people in the LECZ was significantly lower than in most other continents in the year 2000 (24 million or 3.7% of the global LECZ population). Compared to Europe, coastal growth is expected to be higher in Northern America with rates of up to 1.2% (2000–2030), dropping to 0.8% in the decades thereafter (2030–2060), while Europe shows growth rates of 0.3% to 0.1%, respectively (scenario C). The Northern American LECZ population is growing faster than the Latin American one and by 2060 up to 46 million people could be living in the LECZ of Northern America (S2 Table). The U.S. had the largest share of coastal population with 23 million in 2000, rising to 44 million in 2060 (scenario C), ranking eighth among LECZ countries in both years (Table 6 and Table 7). Canada, despite having a much larger LECZ, is sparsely populated along its long northern coastline. Here, a maximum of 1.6 million people could be living below 10 m of elevation by 2060. An interesting feature of the Northern American LECZ is the high number of people in dense urban areas, which reached already almost 60% in 2000 (Table 4).

In **Latin America and the Caribbean,** the LECZ area is about half the size of the Asian LECZ with 424 thousand km^2^ in total, whereas the LECZ population was only about 7% (32 million) of that in Asia in the year 2000. **South America** (Argentina, Brazil, Chile, Colombia, Ecuador, Falkland Islands/Malvinas, French Guiana, Guyana, Suriname, Uruguay, Venezuela) contributed the largest share of coastal population in the year 2000 and is also expected to do so in future: Starting from 22 million in the year 2000, the population in the LECZ could reach between 28 million (scenario B) and 38 million (scenario C) by 2060. In this region, Brazil and Argentina are the two nations with the highest number of people in the LECZ, both in the year 2000 and in future projections (Table 6 and Table 7). In **Brazil**, 12 million people were living in the LECZ (1.4% of the land area) in the year 2000, corresponding to 6.6% of its total population (Table 6). At the same time **Argentina** had about 3.6 million people living the LECZ (about 1.9% of the land area). By 2060, the LECZ population of the two nations could grow to 19 million (Brazil) and 7.6 million (Argentina) (Table 7).

The smallest portion of the global LECZ population is found in **Oceania**. In the year 2000, the LECZ population amounted to 0.5% of the global LECZ population (Table 4; S1 Table). However, this represents at least 11% of the total population of the region, making the proportion higher compared to other regions. Most of these people were living in the LECZ of Australia and New Zealand (2.7 million or 80% of Oceania’s LECZ population in 2000). Growth is projected to be comparatively low in Oceania and could lead to LECZ population totals between 5.0 million and 6.1 million people by 2060 (Scenarios B and C respectively; Table 4). We must note that the results for Oceania do not include data for Tokelau (total population in 2000 [48]: 1,552), Pitcairn (included in Polynesia in the UN data [48], but no separate population records) and for the Federated State of Micronesia (total population in 2000 [48]: 107,103), both for the LECZ and the flood plain analysis. This is due to missing information in the employed data sets, as explained in the section Uncertainties, limitations and evaluation of results. Nevertheless, although highly significant for the respective nations, these numbers would have no major impact on our results at continental or global scale.

### People in the 100-year flood plain in 2000, 2030 and 2060

Our results show that about one third (30%; 189 million) of the global LECZ population was living in the 100-year flood plain in the year 2000 (see Table 5 and Table 8; S3 Table). The number of people at risk from coastal flooding could reach between 268 million and 286 million in 2030, globally (scenarios B and C, respectively). By 2060, up to 411 million people could be affected by extreme flooding events (Scenario C). However, large regional variations exist.

**Asia** had the highest number of people living in the flood plain: 30% (137 million) of Asia’s LECZ population resided in the 100-year flood plain in the baseline year 2000, which made 73% of the total global flood plain population. Our results suggest a rapid population growth for the flood plain population in Asia to between 200 million and 213 million people by 2030 (scenarios B and C; Table 5 and Table 8). By 2060, this number could range between 232 million (scenario B) and 310 million (scenario C), despite slowing growth rates. **Africa**, at the same time, could experience a two-fold increase from 13 million in 2000 to 26 million by 2030 and a further growth to 49 million people in the flood plain by 2060 (scenario C; Table 5 and Table 8; S3 Table).

Europe and Northern America are expected to exhibit a relatively moderate increase (Table 5 and Table 8). In **Europe**, 56% of the LECZ population (28 million people) lived within the 100-year flood plain in the year 2000. The exposed population could grow by 3 million between 2000 and 2030 and an additional 1.2 million by 2060 to reach 32.4 million under scenario D. Scenario D proved to be the highest-end-of-growth scenario for “richer economies”, which is due to the underlying assumptions made in the scenarios (see Table 1). In **Northern America**, the number of people in the flood plain could increase from 4.2 million (year 2000) to about 8.0 million by 2060 (scenario D), with the United States being the country with the largest share of exposed population (Table 5 and Table 8; S4 Table). In **Latin America and the Caribbean**, more than a quarter (19%; 6 million) of the people living in the LECZ were located within the 100-year flood plain in the year 2000. The proportion will remain stable in future, but the total number will reach up to 11 million people in the flood plain by 2060 (scenario C).

According to our results, **Oceania** only has a minor contribution to the global total of people exposed to 1-in-100 year flood events, both in the baseline year 2000 and in the future. However, since Oceania partly consists of a large number of small island states, the impacts of sea-level rise and increasing storm surge heights will affect a large portion of these countries’ inhabitants, as a high percentage of their population and infrastructure is concentrated within a few kilometres of the coast [58]. By 2060, at least 1.6 million people could be at risk from flooding, an increase of up to 100% compared to the year 2000, with more than one third of these people being citizens of small island nations.

## Discussion

### Coastal population development and aspects of coastal migration

Our projections show that, even under the lowest growth assumptions, the global LECZ population could rise by more than 50% between the baseline year 2000 and 2030 (scenario B), from 625 million to 880 million; by 2060, more than a billion people worldwide could be living in the LECZ. Under scenario C the world would face an overall high population growth due to stagnant economic development and exclusive social, political and economic governance (see Fig. 1 and Table 1). In this scenario, the global LECZ would bear 763 million additional people by 2060, compared to the situation in the year 2000, which would be an increase of 122%. For the same scenario between 315 million and 411 million people would be living in the 100-year flood plain by 2060, compared to 189 million in the year 2000. It must be noted that considering for subsidence in deltaic areas and in cities prone to subsidence due to drainage and groundwater pumping would further enhance these numbers [59, 60]. However, this factor was not considered in the present study.

**Fig 5:**
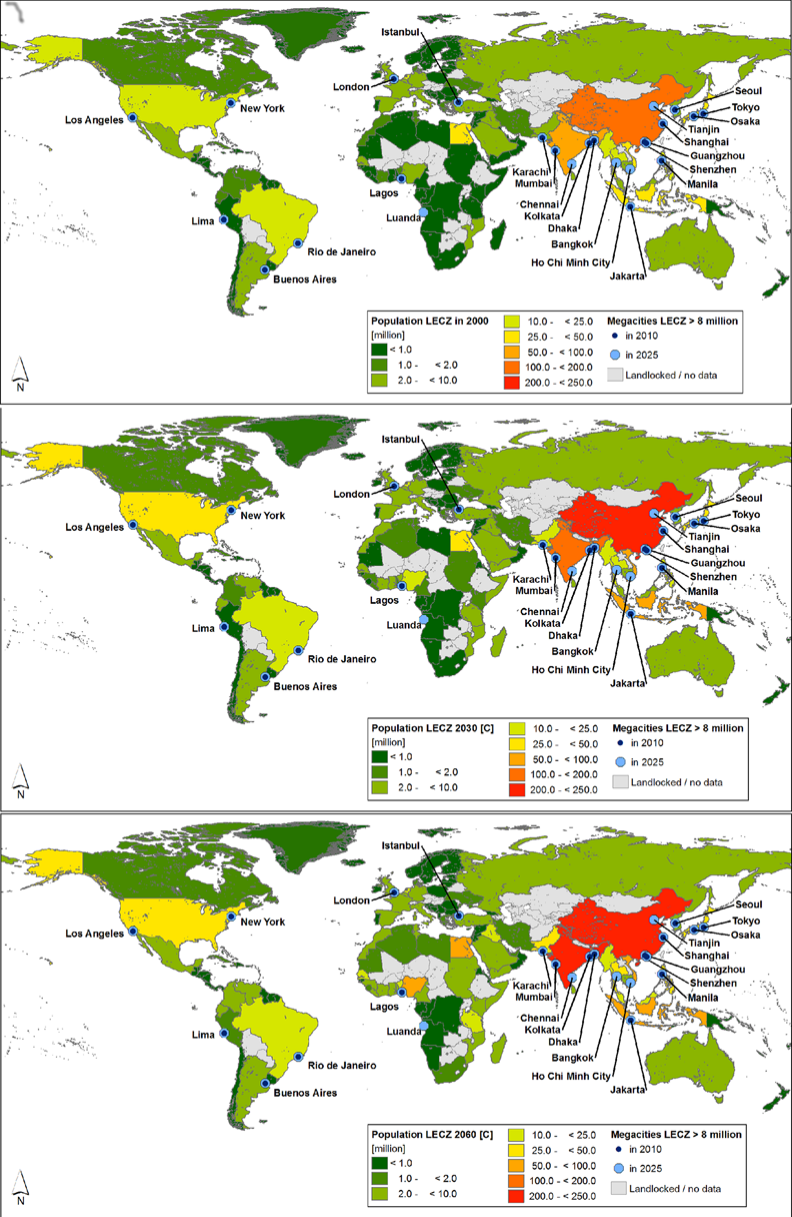
LECZ Population in the year 2000 and for 2030/2060 per country, scenario C. Population estimates (year 2010) and projections (year 2025) for selected megacities (> 8 million people) located in the LECZ were derived from the UN’s World Urbanization Prospects [79].

The results also demonstrate that the less developed countries outnumber the more developed regions in terms of population in the LECZ and in the flood plain, with Asia having had the highest land area, total number of people and urban population in the LECZ in the year 2000 and prevailing in the future (Fig. 5). In Africa, we see a rapid coastal development in terms of overall population growth and urbanisation, which will exacerbate the already high vulnerability of many African coastal countries [33]. By 2060, Egypt and Nigeria are expected to rank in the top ten countries globally, following directly the five Asian countries with the highest exposure: China, India, Bangladesh, Indonesia and Viet Nam. Hanson et al. [18] identified twelve port cities located in these Asian coastal countries to be among the top 20 of the world’s large port cities exposed to 100-year flood levels by 2070 in terms of population. In an assessment of 136 coastal cities by Hallegatte et al. [25], several of these cities were also rated as being highly vulnerable in terms of expected annual damages (flood risk) in 2005 as well as under future scenarios (2050). However, Hanson et al. [18] found 40 million people in urban locations in the 100-year flood plain, considering all coastal cities with more than one million people in 2005. Comparing these figures to our total flood plain population estimates of 189 million (in the year 2000) suggests that most of the flood plain population is actually located in smaller coastal cities, less densely populated urban areas and rural settings.

Nevertheless, among the 25 countries we project to have the largest portion of people in the LECZ in 2060, there are also several developed countries, including the United States of America. The U.S. was already among the 25 countries with the highest LECZ population in the year 2000. Due to the large number of people living in the LECZ (23 million in 2000) and the fact that 61% of these were located in dense urban areas, the U.S. exhibit a relatively strong growth of the total LECZ population in comparison to other developed countries. The U.S. recently encountered major coastal disasters with the Hurricanes Katrina in 2005 and Sandy in 2012, indicating the—possibly increasing—vulnerability and risks associated with settling in low-lying coastal areas of the U.S. [20, 25, 61].

Our projections reflect the scenario assumptions made concerning the socio-economic development pathways of the coastal regions and coastal migration, as well as the underlying low, medium and high growth variants of the UN’s population prospects (see Fig. 1 and Table 1). Scenario B with its lowest-end-of-growth assumptions (10^th^ percentile or low growth variant) produces the lowest projections of coastal growth, despite a coastal correction factor of 2.0 assigned to coastal urban areas in the developing world to account for massive migration to regional growth poles. The scenarios A and C project the highest population growth in the LECZ for the “less developed regions”. Nevertheless, assumptions of increased migration from poorer countries to richer countries in combination with a high population growth variant for the developing world (90^th^ percentile or high growth variant) in scenario C result in overall higher coastal growth compared to scenario A. In this scenario, we translated the assumed patterns of more rapid internal migration in faster growing developing countries into slightly higher coastal urban growth, while coastal non-urban growth is reduced due to stagnant economy and migration to regional growth poles in comparison to scenario A. Only Africa exhibits a different behaviour in the period between 2000 and 2030 with strongest growth under scenario A. This is explained by a high percentage of non-urban coastal population in the African countries and the assumption that developing countries partially experience rapid coastal migration with expansive urban growth. In contrast to this, the “richer economies” in Europe, Northern America, Japan and Australia/New Zealand would face the highest coastal growth under scenario D. Although in this scenario inclusive governance is assumed to keep the global population growth at the low end of forecasts (50^th^ percentile or medium growth variant), richer economies exhibit relatively strong coastal growth due to an increased demand for migrants to fill in the labour market for the aging population [39]. It has to be noted, though, that due to the methodology employed, we cannot explicitly differentiate between urban and non-urban population numbers in our projections, as the latter also include a certain proportion of urban population. This is due to the fact that we did not account spatially for transitions between dense urban, suburban and rural areas. However, these transitions are considered implicitly through our assumptions of coastal urban growth. We are therefore confident that the total numbers produced in this study constitute reliable projections of people in the LECZ and in the 100-year flood plain.

Net migration from developing to developed countries, as well as assumptions on fertility, are inherently included in the employed UN’s population prospect variants [46]. General effects of environmental pressures and disasters on migration are considered in the Foresight Project’s socio-economic scenarios [39]. However, possible out-migration and displacement as a response to increased flood risks or inundation was not considered spatially in our assessment. More explicit consideration of these factors in future work is important, especially when considering that the areas at risk, i.e. coastal flood plains and deltaic areas, are at the same time a “major migrant destinations since they offer better economic opportunities through their concentration of industry and services” [62]. The UK’s Government Office for Science [38] concludes that environmental change in the LECZ, such as sea-level rise and increasing occurrence extreme events, will affect the existing structural drivers of migration through the induced socio-economic impacts. However, as Black et al. [63] and Warner [64] point out, the factors that drive environmental migration are complex and multi-layered, and migration as well as displacement are some of the possible responses. The role of adaptation to coastal flooding and sea-level rise will also need to be considered [16, 21, 25, 65]. Curtis and Schneider [66] stress that migration networks between coastal and inland areas or between inundated and not-inundated coastal counties may be another essential factor to account for when assessing future coastal population. Socio-demographic, economic and environmental characteristics as well as the political setting of a coastal area or region determine the response to coastal hazards. Yet, such a level of detail is hard to achieve in global to regional scale studies.

### Uncertainties, limitations and evaluation of results

Our estimates of total land area and population in the LECZ for the year 2000 are in agreement with the findings of previous studies [1, 5], with deviations being in the order of 4% for the global total and between 1% and 10% when comparing continental totals (see Table 9). However, our assessments suggest a significantly smaller proportion of urban population within the LECZ. This deviation can be explained by the different data used for the identification of urban areas and the resulting differences in the definitions of “urban”. While McGranahan et al. [5] and Balk et al. [1] used the urban extent grids of the Global Rural-Urban Mapping Project GRUMP (GRUMP alpha), we employed the higher resolution MODIS 500-m Map of Global Urban Extent (see Material and Methods; Table 2). This decision was based on the work of Potere and Schneider [67], Schneider et al. [42] and Seto et al. [7] who found GRUMP to overestimate urban land in comparison to other global urban maps and the MODIS 500-m map to have the highest overall accuracy [42, 67]. In addition, we conducted extensive visual checks of urban areas to compare their representation in both data sets, also using satellite imagery for validation (Google Earth; ArcGIS World Imagery). For most regions, the urban extent of the MODIS data set appeared to be considerably more representative of built-up urban areas than GRUMP. The latter seems to overestimate urban extent and city size but captures other types of settlements such as urban slums, which the MODIS grid excludes. We also observed that both MODIS and GRUMP urban extent grids are likely to include non-residential built-up areas such as industrial districts or commercial centres. At the same time, by using the MODIS urban extent grid in combination with the GRUMP population count grid to approximate urban population, specific types of possibly densely populated residential areas within urban administrative units, such as informal settlements and urban slums, might have been classified as non-urban population in our assessment.

**Table 9:** Comparison of different studies estimating the LECZ land area and population for the year 2000.

Region	Study	Employed land use data	Total area LECZ [km^2^]	Total pop. LECZ [million]	Urban pop. LECZ [million]
**Global**	This study	MODIS-500m[42, 43]	2.598.623	625.2	146.9
	McGranahan et al. [5]	GRUMP alpha [84]	2.700.000	634.0	360.0
**Africa**	This study	MODIS-500m [42, 43]	193.658	54.2	8.9
	McGranahan et al. [5]	GRUMP alpha [84]	191.000	56.0	31.0
	Balk et al. [1]	GRUMP alpha [84]	NA	NA	31.5
**Asia**	This study	MODIS-500m [42, 43]	859.215	460.8	92.8
	McGranahan et al. [5]	GRUMP alpha [84]	881.000	466.0	238.0
	Balk et al. [1]	GRUMP alpha [84]	NA	NA	253.7
**Latin America**	This study	MODIS-500m [42, 43]	423.863	32.2	9.3
	McGranahan et al. [5]	GRUMP alpha [84]	397.000	29.0	23.0
	Balk et al. [1]	GRUMP alpha [84]	NA	NA	17.7
**India**	This study	MODIS-500m [42, 43]	82.262	63.9	10.5
	McGranahan et al. [5]	GRUMP alpha [84]	NA	63.2	NA
	Balk et al. [1]	GRUMP alpha [84]	NA	NA	37.3

**Abbreviations**: pop. = population.

Further uncertainties may have been introduced when combining the MODIS urban extent data [42, 43] with the GRUMP population data [44], where resampling may have led to incorrect allocation of population into urban and non-urban classes. These uncertainties could not be quantified in the context of this work, but we expect them to have only minor influence on the population figures. Overall we are confident to have produced representative global estimates of LECZ population, though we have to stress that our urban population refers to people living in dense urban areas (see Material and Methods). We may underestimate urban population for less densely built-up urban areas, for cities with large vegetated areas or for urban settlements in less developed countries with structures that resemble rural areas, such as dirt roads. For this reason our baseline estimates of urban population are likely to be at the lower bound for the year 2000, compared to e.g. the results of McGranahan et al. [5] and Balk et al. [1].

As discussed by Balk et al. [68], amongst others, there are further issues related to the criteria and methods whereby populations and the respective areas are identified as urban or non-urban in spatial data and census data. For census data, there is no common set of criteria and definitions for classifying urban and non-urban (or rural) population between countries [69, 70]. In a similar way, spatial population and urban extent data are also based on specific (but possibly different) criteria and methods for differentiating between urban and non-urban areas and for spatially allocating people [42, 43, 67, 70]. These issues need to be considered when combining spatial population and urban extent data with census-based data. Nevertheless, we are confident that by combining spatial and non-spatial population data we did not introduce additional uncertainty. The UN’s population and urbanisation data were used to derive annual rates of coastal urban and non-urban growth, as explained in Material and Methods. These rates were then applied to the mapped urban and non-urban baseline population shares.

As a result of the resolution and scale of this analysis, some issues with small coastal countries occurred, such as missing information and mis-registration issues between spatial data layers. This became particularly evident when analysing data of small islands and island states in this global approach. Several of these could not be considered in this study because of missing information in the GRUMP population count grid [44] (St. Helena, French Southern Territories, Tokelau and Pitcairn Islands) and in the land area data set [71] (Norfolk Island and the Federated State of Micronesia). In the flood plain analysis we identified spatial mis-matches between the GRUMP data sets [44, 71] and the more detailed GADM boundaries [72]. Similar issues due to mismatches between elevation and population data sets had been reported by McGranahan et al. [5] and Lichter et al [6].

Nevertheless, despite addressing those mis-matches (see Material and Methods), we may still underestimate the number of people in the flood plain. For instance, we estimated 189 million people to have been living in the 100-year flood plain in the year 2000, globally, while Jongman et al. [73] estimated 271 million people exposed to 1-in-100-year coastal flood events in 2010. They projected 345 million people to be living in the 100-year flood plain in 2050, based on the Medium Fertility projections of the United Nations’ 2006 Revision of the World Population Prospects, while our results suggest a coastal growth to 340 million people by 2060 under a medium growth variant (scenario D). Although these numbers do compare well, we must note that there is a difference of ten years between the baseline years and the projections and that Jongman et al. [73] did not account for upward displacement of the flood plain from sea-level rise. The observed differences between their study and our assessment can further result from variations in the base data employed: Jongman et al. [73] used a finer resolution SRTM grid at 3 arc sec resolution but coarser resolution population density data at 5 arc min resolution and, as mentioned earlier, an older version of the UN’s demographic data.

The issues discussed above constitute inherent characteristics of analysis that integrate global data sets from different sources, as discussed by several authors [6, 27, 56, 68, 74]. Despite these common uncertainties and limitations, we are confident that our results present improved first order estimates of the population development and exposure of land and people in coastal regions. These estimates can provide a reliable basis for exploring and comparing future development trends and pathways at regional, continental and global levels. However, we also see scope for improvement regarding the differential projection of urban and non-urban population in the coastal zone. The use of dynamic spatial models of land-use change in the analysis would allow for explicit consideration of the expansive dimension of urban growth and the spatial transitions between different land use categories. Such a model could then be combined with more detailed scenarios and country-specific coastal correction factors to spatially differentiate between urban growth in density, urban expansion including peri-urbanisation and rural population change.

However, as outlined above, the categorisation of urban and non-urban (or rural) areas and populations currently suffers from a lack of unambiguous and consistent definitions of the respective classes, or other forms of land use and settlement structures, and their representation in global land use/land cover maps, population maps and census data. Thus, looking at the importance of global data sets for assessing global- and climate-change related impacts and with the encountered limitations and uncertainties in mind, we strongly support Mondal and Tatem [35] in their pleading for “spatial population datasets built on accurate, contemporary and detailed census data”. In fact, there is an urgent need for a more detailed approximation of population and settlement structures. These could possibly be based upon existing data models such as GRUMP and MODIS for improved and consistent global population and land use data. Further, we recommend detailed explorations of both data sets with respect to capturing settlements of different types and the respective population shares, for example introducing a third class of peri-urban and comparing different combinations of global urban extent data and population data. Also, when analysing the future flood plain population, the role of subsidence should be considered in addition to sea-level rise. Finally, this first-order assessment could also be improved in future studies by accounting for migration and displacement due to environmental changes and climate change-related effects such as sea-level rise. Yet, this would require employing other spatial assessment methods in order to relocate people from the flood plain and consider migration networks, as discussed by Curtis and Schneider [66].

As outlined above, our results are based on a series of assumptions (e.g. with regard to coastal growth) and data sets (e.g. MODIS urban extent data, GRUMP population count data and the UN’s 2009 and 2010 urbanisation and population data), and the overall assessment is confined by certain limitations and uncertainties. We recommend that continued studies on this topic are needed. By employing more recent or improved data and refining methods and scenarios or accounting for the discussed uncertainties and limitations, the results will inevitably evolve. For example, new population projections and scenarios come to different conclusions whether population growth will level off before 2100 [75, 76] or continue to grow [77] and how population will change in China or in fast-growing countries of Africa. But for the time being, our assessment represents plausible scenarios of future population exposure in coastal zones.

## Summary and Conclusions

This study has produced new estimates of the number of people living in the low-elevation coastal zones (LECZ) and the 100-year flood plain. We have constructed plausible futures of the LECZ population and of people in the flood plain in 2030 and 2060 and highlighted regions of high exposure. These estimates are based on a series of scenario-dependent assumptions on climate change effects relating to sea-level rise, future socio-economic development and coastal migration and are more detailed than previous work. The population projections for the LECZ and the coastal flood plain are, to our knowledge, the only quantitative global estimates that account for (i) the faster growth of coastal regions in comparison to the landlocked hinterland and (ii) differential population growth of coastal urban areas as opposed to coastal non-urban areas.

The results show significant increases in coastal population living in the LECZ and of people being potentially exposed to coastal flood events. They highlight regions that will most likely experience rapid increases in exposure, such as Africa, and depict that Asia is the continent that has had the largest number of total and of urban population in the LECZ and the 100-year flood plain in the year 2000 and will continue to do so in the future. Our results emphasise that less developed countries are more exposed to flooding than more developed regions. Africa and Asia are expected to become increasingly exposed to sea-level rise and coastal hazards and thereby many countries that already now experience high vulnerability to such hazards. The five Asian countries China, India, Bangladesh, Indonesia and Viet Nam accounted for more than half of the global LECZ population in the year 2000 and will continue to do so under future scenarios, despite the rapid coastal growth of several African coastal nations. Further, our study suggests that densely-populated urban areas are less prevalent in the LECZ than expected, as our baseline assessment produced a significantly smaller urban population than previous studies. We need to stress, however, that earlier studies relate ‘urban’ areas to urban agglomerations that encompass densely populated urban areas and suburban and even peri-urban areas population. This is a topic for further investigation.

Our assessments provide useful information for better understanding future coastal development and exposure to coastal flooding and submergence at global, regional and national scales. Further, they can be used as inputs to impact models for different scenarios of change. These new projections of coastal population build ground for further analyses beyond the scope of the study presented here. These could, for example, consider the spatial dynamics of urbanisation, the current limitations and inconsistencies related to global data sets or the interactions and feedbacks between environmental change and migration. One aspect rarely discussed, but strongly related to the theme of environmental migration, is a possible reversion of the coastward migration trend due to increasing impacts from climate change, subsidence and extreme events. Furthermore, considering adaptation and mitigation processes would allow for a more in-depth analysis of the actual exposure, vulnerability and risk of coastal nations and regions. Hence, further research is required to better understand the human-environment interactions in coastal regions, improve forecasts of impacts and responses for a better management of coastal change and to build resilient and sustainable coastal communities now and into the future [78].

## Supporting Information

S1 Data (XLSX)

S1 Table Land area and population globally, of coastal countries and in the LECZ, baseline year 2000, per development status, continent and region. (DOCX)

S2 Table Population in the LECZ projected for 2030 and 2060, scenarios A-D, per development status, continent and region. (DOCX)

S3 Table People in the 100-year flood plain in 2000 and projected to 2030 and 2060, scenarios A-D, per development status, continent and region. (DOCX)

S4 Table Demographic base data and assessment results per region and reporting unit (countries). (XLSX)
