# Correction: Future Coastal Population Growth and Exposure to Sea-Level Rise and Coastal Flooding - A Global Assessment

**DOI:** 10.1371/journal.pone.0131375

**Published:** 2015-06-18

**Authors:** The PLOS ONE Staff

## Notice of Republication

This article was republished on May 29, 2015, to correct formatting errors that were introduced during the typesetting process. Specifically, within the Materials and Methods section, "People in the 100-year flood plain" appeared as a subsection of "Land area and population in the LECZ". "People in the 100-year flood plain" has now been reinstated as a level-2 heading within the Materials and Methods section. Additionally, the Results section was not linked from the left-hand sidebar detailing the sections of the paper. The Results section has now been linked from the sidebar. Finally, the Supporting Information file "S1 Data" was a duplicate of "S4 Table", and should not have been published. This file has been removed. The publisher apologizes for these errors.
