# Supplementary material for: Future Coastal Population Growth and Exposure to Sea-Level Rise and Coastal Flooding - A Global Assessment
Source: PLoS One. 2015 Mar 11;10(3):e0118571. doi: 10.1371/journal.pone.0118571 (PMC4367969; doi:10.1371/journal.pone.0118571)
Supplement: S1 Table — (DOCX) [file pone.0118571.s002.docx]

Table S2: Population in the LECZ projected for 2030 and 2060, scenarios A-D, per development status, continent and region.

| **Region** | **Baseline year 2000** | | **Scenario A** | | **Scenario B** | | **Scenario C** | | **Scenario D** | |
| --- | --- | --- | --- | --- | --- | --- | --- | --- | --- | --- |
|  | Total 2000 [million] | LECZ 2000 [million] | LECZ 2030 [million] | LECZ 2060 [million] | LECZ 2030 [million] | LECZ 2060 [million] | LECZ 2030 [million] | LECZ 2060 [million] | LECZ 2030 [million] | LECZ 2060 [million] |
| **World** | **6,100.8** | **625.2** | **938.9** | **1,318.3** | **879.1** | **1,052.8** | **948.9** | **1,388.2** | **892.9** | **1,128.1** |
| - More dev. regions | 1,188.8 | 107.5 | 120.6 | 124.1 | 120.6 | 124.1 | 125.8 | 138.4 | 125.9 | 138.4 |
| - Less dev. regions including least dev. countries | 4,912.0 | 517.7 | 818.4 | 1,194.1 | 758.6 | 928.6 | 823.1 | 1,249.8 | 767.1 | 989.7 |
| - Least dev. countries | 662.0 | 93.0 | 146.9 | 231.4 | 132.5 | 181.9 | 146.5 | 242.0 | 136.3 | 192.7 |
| - Less dev. regions, excluding least dev. countries | 4,250.0 | 424.7 | 671.5 | 962.8 | 626.1 | 746.7 | 676.6 | 1,007.7 | 630.7 | 797.0 |
| - Less dev. regions, excluding China | 3,642.9 | 373.7 | 619.3 | 958.8 | 561.4 | 729.1 | 619.0 | 1,005.0 | 574.6 | 785.5 |
| - China | 1,269.1 | 144.0 | 199.0 | 235.4 | 197.2 | 199.6 | 204.1 | 244.8 | 192.4 | 204.2 |
| - Sub-Saharan Africa | 669.1 | 24.2 | 66.4 | 160.0 | 63.1 | 136.5 | 65.7 | 174.0 | 61.3 | 126.6 |
| **AFRICA** | **811.1** | **54.2** | **117.6** | **229.3** | **108.5** | **190.0** | **116.8** | **245.2** | **108.9** | **185.6** |
| Eastern Africa | 251.6 | 5.2 | 15.1 | 39.9 | 13.8 | 34.8 | 14.1 | 42.5 | 13.8 | 31.1 |
| Middle Africa | 96.2 | 1.1 | 2.2 | 3.8 | 2.0 | 3.0 | 2.2 | 4.1 | 2.0 | 3.0 |
| Northern Africa | 176.2 | 30.3 | 52.3 | 72.4 | 46.6 | 56.3 | 52.3 | 74.8 | 48.6 | 61.4 |
| Southern Africa | 51.4 | .5 | .9 | 1.5 | .8 | .9 | 1.0 | 1.7 | .9 | 1.1 |
| Western Africa | 235.7 | 17.1 | 47.1 | 111.7 | 45.3 | 95.0 | 47.2 | 122.3 | 43.6 | 88.9 |
| **ASIA** | **3,697.1** | **460.8** | **688.7** | **943.9** | **640.3** | **728.6** | **695.0** | **983.3** | **649.4** | **792.8** |
| Eastern Asia | 1,473.3 | 180.9 | 238.1 | 276.3 | 235.6 | 238.7 | 244.5 | 287.2 | 232.4 | 245.3 |
| South-Central Asia | 1,515.6 | 135.7 | 225.5 | 345.2 | 196.5 | 254.1 | 224.0 | 362.9 | 205.6 | 281.3 |
| South-Eastern Asia | 523.8 | 133.2 | 197.6 | 278.2 | 182.6 | 201.6 | 199.5 | 287.6 | 185.7 | 230.3 |
| Western Asia | 184.4 | 11.1 | 27.5 | 44.2 | 25.6 | 34.3 | 27.0 | 45.6 | 25.7 | 35.8 |
| **EUROPE** | **726.8** | **50.0** | **52.8** | **52.1** | **52.8** | **52.1** | **54.5** | **55.7** | **54.5** | **55.7** |
| Eastern Europe | 304.2 | 6.8 | 6.9 | 7.0 | 6.9 | 7.0 | 6.9 | 7.0 | 6.9 | 7.0 |
| Northern Europe | 94.3 | 11.2 | 11.9 | 11.6 | 11.9 | 11.6 | 12.3 | 12.8 | 12.3 | 12.8 |
| Southern Europe | 145.1 | 10.6 | 11.7 | 11.8 | 11.7 | 11.8 | 12.0 | 12.6 | 12.0 | 12.6 |
| Western Europe | 183.1 | 21.4 | 22.2 | 21.7 | 22.2 | 21.7 | 23.2 | 23.3 | 23.2 | 23.3 |
| **LATIN AMERICA AND THE CARIBBEAN** | **521.4** | **32.2** | **41.7** | **50.6** | **39.5** | **40.1** | **42.3** | **52.3** | **39.8** | **42.6** |
| Caribbean | 38.4 | 3.5 | 4.3 | 5.4 | 4.1 | 4.4 | 4.4 | 5.6 | 4.1 | 4.7 |
| Central America | 135.6 | 6.8 | 7.9 | 8.8 | 7.6 | 7.8 | 7.9 | 8.9 | 7.6 | 8.0 |
| South America | 347.4 | 21.9 | 29.5 | 36.4 | 27.7 | 27.9 | 30.1 | 37.7 | 28.0 | 29.8 |
| **NORTHERN AMERICA** | **313.3** | **24.6** | **33.5** | **37.0** | **33.5** | **37.0** | **35.5** | **45.5** | **35.5** | **45.5** |
| **OCEANIA** | **31.1** | **3.3** | **4.7** | **5.5** | **4.6** | **5.0** | **4.8** | **6.1** | **4.8** | **5.8** |
| Australia/New Zealand | 23.0 | 2.7 | 3.5 | 3.8 | 3.5 | 3.8 | 3.7 | 4.4 | 3.7 | 4.4 |
| Melanesia | 7.0 | 0.4 | 0.8 | 1.2 | 0.7 | 0.9 | 0.7 | 1.1 | 0.7 | 1.0 |
| Micronesia | 0.5 | 0.2 | 0.3 | 0.3 | 0.2 | 0.2 | 0.3 | 0.3 | 0.2 | 0.3 |
| Polynesia | 0.6 | 0.1 | 0.2 | 0.2 | 0.1 | 0.1 | 0.2 | 0.2 | 0.2 | 0.2 |

**Total population** is based on [1]. Classifications by major region and develoment status follow the UN classification scheme [2, 3]. All LECZ areas and population numbers are based on own assessments. **Abbreviations**: dev. = developed.

**References**

1. United Nations. World Population Prospects: The 2010 Revision. File 1: Total population (both sexes combined) by major area, region and country, annually for 1950-2100 (thousands). Database. New York: United Nations, Department of Economic and Social Affairs, Population Division; 2011. Available: <http://esa.un.org/wpp/Excel-Data/DB02_Stock_Indicators/WPP2010_DB2_F01_TOTAL_POPULATION_BOTH_SEXES.XLS>. Accessed 27 June 2011.
2. United Nations. World Population Prospects: The 2010 Revision, Highlights and Advance Tables. Working Paper No ESA/P/WP220. New York: Department of Economic and Social Affairs, Population Division; 2011. Available: <http://esa.un.org/unpd/wpp/Documentation/pdf/WPP2010_Highlights.pdf>. Accessed 29 June 2011.
3. United Nations. World Population Prospects: The 2010 Revision. File 0-1: Location list with codes, description, major area, region and development group, countries with explicit HIV/AIDS mortality modelling in WPP 2010 revision, HIV prevalence rate (%) in population aged 15–49 years in 2009 (UNAIDS, 2011) and by prevalence group. Database. New York: United Nations, Department of Economic and Social Affairs, Population Division; 2011. Available: <http://esa.un.org/unpd/wpp/Excel-Data/WPP2010_F01_LOCATIONS.XLS>. Accessed 29 June 2011.
