# Supplementary material for: Future Coastal Population Growth and Exposure to Sea-Level Rise and Coastal Flooding - A Global Assessment
Source: PLoS One. 2015 Mar 11;10(3):e0118571. doi: 10.1371/journal.pone.0118571 (PMC4367969; doi:10.1371/journal.pone.0118571)
Supplement: S2 Table — (DOCX) [file pone.0118571.s003.docx]

Table S3: People in the 100-year flood plain in 2000 and projected to 2030 and 2060, scenarios A-D, per development status, continent and region.

| **Region** | **Total population** | **LECZ population** | **People in the 100-year flood plain** | | | | | | | | | |
| --- | --- | --- | --- | --- | --- | --- | --- | --- | --- | --- | --- | --- |
|  | **2000** | **2000** | **2000** | | **2030** | | | | **2060** | | | |
|  | Baseline [million] | Baseline [million] | Baseline [million] | % of global flood pl. 2000 | Scenario A [million] | Scenario B [million] | Scenario C [million] | Scenario D [million] | Scenario A [million] | Scenario B [million] | Scenario C [million] | Scenario D [million] |
| **WORLD** | **6,100.8** | **625.2** | **189.2** | **100.0** | **282.2** | **268.1** | **285.9** | **271.0** | **392.9** | **315.5** | **411.3** | **339.5** |
| More developed regions | 1,188.8 | 107.5 | 41.2 | 21.8 | 45.4 | 45.4 | 47.3 | 47.3 | 46.9 | 46.9 | 51.0 | 51.0 |
| Less developed regions, including least developed countries | 4,912.0 | 517.7 | 147.9 | 78.2 | 236.8 | 222.6 | 238.6 | 223.7 | 346.0 | 268.6 | 360.3 | 288.4 |
| Least developed countries | 662.0 | 93.0 | 12.6 | 6.7 | 22.9 | 20.9 | 22.6 | 21.3 | 41.6 | 33.8 | 43.8 | 34.2 |
| Less developed regions, excluding least developed countries | 4,250.0 | 424.7 | 135.3 | 71.5 | 213.9 | 201.7 | 215.9 | 202.5 | 304.5 | 234.8 | 316.5 | 254.2 |
| Less developed regions, excluding China | 3,642.9 | 373.7 | 91.9 | 48.6 | 156.0 | 142.6 | 155.7 | 145.6 | 246.7 | 184.4 | 256.9 | 202.2 |
| China | 1,269.1 | 144.0 | 56.0 | 29.6 | 56.0 | 80.8 | 80.0 | 82.8 | 78.1 | 99.4 | 84.3 | 103.4 |
| Sub-Saharan Africa | 669.1 | 24.2 | 3.4 | 1.8 | 9.6 | 9.0 | 9.4 | 8.9 | 23.6 | 20.0 | 25.3 | 18.6 |
| **AFRICA** | **811.1** | **54.2** | **12.6** | **6.7** | **26.0** | **23.5** | **25.7** | **24.1** | **46.9** | **37.9** | **49.2** | **38.4** |
| Eastern Africa | 251.6 | 5.2 | 1.4 | 0.8 | 4.3 | 4.0 | 4.0 | 3.9 | 11.8 | 10.4 | 12.7 | 9.3 |
| Middle Africa | 96.2 | 1.1 | 0.2 | 0.1 | 0.3 | 0.3 | 0.3 | 0.3 | 0.5 | 0.4 | 0.5 | 0.4 |
| Northern Africa | 176.2 | 30.3 | 9.2 | 4.9 | 16.3 | 14.6 | 16.3 | 15.2 | 23.3 | 18.1 | 24.0 | 19.9 |
| Southern Africa | 51.4 | 0.5 | 0.1 | 0.1 | 0.2 | 0.2 | 0.2 | 0.2 | 0.3 | 0.2 | 0.3 | 0.2 |
| Western Africa | 235.7 | 17.1 | 1.7 | 0.9 | 4.8 | 4.5 | 4.8 | 4.5 | 11.0 | 8.9 | 11.7 | 8.7 |
| **ASIA** | **3,697.1** | **460.8** | **137.3** | **72.6** | **211.1** | **199.9** | **213.4** | **200.7** | **297.6** | **231.6** | **309.6** | **250.7** |
| Eastern Asia | 1,473.3 | 180.9 | 67.2 | 35.5 | 93.4 | 92.3 | 95.8 | 90.9 | 113.3 | 97.2 | 117.7 | 99.9 |
| South-Central Asia | 1,515.6 | 135.7 | 25.0 | 13.2 | 46.9 | 40.7 | 46.3 | 42.6 | 79.2 | 58.2 | 83.5 | 63.8 |
| South-Eastern Asia | 523.8 | 133.2 | 41.4 | 21.9 | 60.3 | 57.1 | 61.0 | 57.4 | 86.4 | 61.8 | 89.0 | 71.9 |
| Western Asia | 184.4 | 11.1 | 3.8 | 2.0 | 10.6 | 9.8 | 10.3 | 9.9 | 18.8 | 14.4 | 19.3 | 15.0 |
| **EUROPE** | **726.8** | **50.0** | **28.2** | **14.9** | **30.1** | **30.1** | **31.2** | **31.2** | **30.2** | **30.2** | **32.4** | **32.4** |
| Eastern Europe | 304.2 | 6.8 | 2.7 | 1.4 | 2.8 | 2.8 | 2.8 | 2.8 | 2.9 | 2.9 | 2.9 | 2.9 |
| Northern Europe | 94.3 | 11.2 | 5.1 | 2.7 | 5.6 | 5.6 | 5.8 | 5.8 | 5.6 | 5.6 | 6.2 | 6.2 |
| Southern Europe | 145.1 | 10.6 | 4.3 | 2.3 | 5.0 | 5.0 | 5.1 | 5.1 | 5.3 | 5.3 | 5.7 | 5.7 |
| Western Europe | 183.1 | 21.4 | 16.1 | 8.5 | 16.7 | 16.7 | 17.5 | 17.5 | 16.4 | 16.4 | 17.6 | 17.6 |
| **LATIN AMERICA AND THE CARIBBEAN** | **521.4** | **32.2** | **6.1** | **3.2** | **8.1** | **7.7** | **8.2** | **7.7** | **10.2** | **7.9** | **10.5** | **8.5** |
| Caribbean | 38.4 | 3.5 | 0.7 | 0.4 | 0.8 | 0.8 | 0.8 | 0.8 | 1.0 | 0.8 | 1.1 | 0.9 |
| Central America | 135.6 | 6.8 | 1.0 | 0.5 | 1.2 | 1.1 | 1.2 | 1.1 | 1.3 | 1.2 | 1.4 | 1.2 |
| South America | 347.4 | 21.9 | 4.4 | 2.3 | 6.1 | 5.8 | 6.2 | 5.8 | 7.8 | 5.9 | 8.1 | 6.4 |
| **NORTHERN AMERICA** | **313.3** | **24.6** | **4.2** | **2.2** | **5.8** | **5.8** | **6.1** | **6.1** | **6.5** | **6.5** | **8.0** | **8.0** |
| **OCEANIA** | **31.1** | **3.3** | **0.8** | **0.4** | **1.2** | **1.2** | **1.2** | **1.2** | **1.5** | **1.3** | **1.6** | **1.5** |
| Australia/New Zealand | 23.0 | 2.7 | 0.5 | 0.3 | 0.7 | 0.7 | 0.8 | 0.8 | 0.8 | 0.8 | 1.0 | 1.0 |
| Melanesia | 7.0 | 0.4 | 0.1 | 0.1 | 0.3 | 0.2 | 0.2 | 0.2 | 0.4 | 0.3 | 0.4 | 0.3 |
| Micronesia | 0.5 | 0.2 | 0.1 | 0.1 | 0.1 | 0.1 | 0.1 | 0.1 | 0.2 | 0.1 | 0.2 | 0.2 |
| Polynesia | 0.6 | 0.1 | 0.1 | 0.0 | 0.1 | 0.1 | 0.1 | 0.1 | 0.1 | 0.1 | 0.1 | 0.1 |

**Total population** is based on [1]. Classifications by major region and develoment status follow the UN classification scheme [2, 3]. All LECZ areas and population numbers are based on own assessments. **Abbreviations**: dev. = developed; flood pl. = flood plain.

**References**

1. United Nations. World Population Prospects: The 2010 Revision. File 1: Total population (both sexes combined) by major area, region and country, annually for 1950-2100 (thousands). Database. New York: United Nations, Department of Economic and Social Affairs, Population Division; 2011. Available: <http://esa.un.org/wpp/Excel-Data/DB02_Stock_Indicators/WPP2010_DB2_F01_TOTAL_POPULATION_BOTH_SEXES.XLS>. Accessed 27 June 2011.
2. United Nations. World Population Prospects: The 2010 Revision, Highlights and Advance Tables. Working Paper No ESA/P/WP220. New York: Department of Economic and Social Affairs, Population Division; 2011. Available: <http://esa.un.org/unpd/wpp/Documentation/pdf/WPP2010_Highlights.pdf>. Accessed 29 June 2011.
3. United Nations. World Population Prospects: The 2010 Revision. File 0-1: Location list with codes, description, major area, region and development group, countries with explicit HIV/AIDS mortality modelling in WPP 2010 revision, HIV prevalence rate (%) in population aged 15–49 years in 2009 (UNAIDS, 2011) and by prevalence group. Database. New York: United Nations, Department of Economic and Social Affairs, Population Division; 2011. Available: <http://esa.un.org/unpd/wpp/Excel-Data/WPP2010_F01_LOCATIONS.XLS>. Accessed 29 June 2011.
